# Supplementary material for: Birth preparedness and complication readiness knowledge, practices and its associated factors among recently delivered women: A cross-sectional study in Bharatpur, Chitwan, Nepal
Source: PLoS One. 2026 Jan 7;21(1):e0340376. doi: 10.1371/journal.pone.0340376 (PMC12779156; doi:10.1371/journal.pone.0340376)
Supplement: Attachment — Submitted filename: PONE-D-24-29050_R1_manuscript for review_Awini.pdf [file pone.0340376.s003.pdf]

PLOS ONE

Birth Preparedness And Complication Readiness Knowledge And Practices Among Recently Delivered Women: A Cross-Sectional Study In Bharatpur, Chitwan, Nepal

--Manuscript Draft--

**Manuscript Number:**	PONE-D-24-29050R1
**Article Type:**	Research Article
**Full Title:**	Birth Preparedness And Complication Readiness Knowledge And Practices Among Recently Delivered Women: A Cross-Sectional Study In Bharatpur, Chitwan, Nepal
**Short Title:**	A cross sectional study on BPCR Knowledge and Practices in Chitwan, Nepal
**Corresponding Author:**	Asmita Ghimire, Bachelor<br>Chitwan Medical College<br>Bharatpur, Bagmati NEPAL
**Keywords:**	BPCR, Birth Preparedness and Complication Readiness, Recently delivered women, Pregnancy, signs and symptoms
**Abstract:**	Background<br><br>Birth preparedness and complication readiness (BPCR) is a comprehensive strategy, aimed at ensuring that expectant mothers and their families proactively planning for normal delivery and preparing for potential complications that may arise during pregnancy, labor, delivery, and the postpartum period without any delays.<br><br>Objective<br><br>This study was conducted to assess birth preparedness and complication readiness knowledge, practices and its associated factors among recently delivered women in Bharatpur city, Chitwan, Nepal.<br><br>Methods<br><br>A community based cross-sectional study was conducted in 2022, on a sample of 220 recently delivered women. Data were collected using pre-tested structured interview schedule. The collected data were analyzed by IBM SPSS 20 version software. Variables with p-value ≤0.05 on the bivariate analysis were in included in multivariate analysis. Adjusted odds ratios (AOR) with the respective 95% Confidence Interval (CI) and a p-value <0.05 was used to set statistically significant variables in the multivariable analysis.<br><br>Results<br><br>Among 220 recently delivered women, the majority of the women identified the place of delivery (91.4%) and saved money for childbirth (97.7%). Similarly, most of them arranged transportation (87.3%), identified a companion (85.9%), and arranged necessary materials (90%) for childbirth. Considerable (52.3%) women identified skilled birth attendants. Preparedness for blood donors (36.4%) found to be low as compared to other components. Overall, 46.8% of recently delivered women were well prepared. Family type, knowledge on components of BPCR, obstetric signs and symptoms and ANC visits were found to be statistically significant (p-value<0.05) with birth preparedness practice. Associated characteristics were further subjected to multivariate logistic regression where knowledge on components of BPCR (AOR=5.34,95%CI: (2.728-10.478) was found significantly associated with BPCR practice.<br><br>Conclusion<br><br>The overall proportion of women who prepared for birth and its complication readiness was found to be higher as compared to other reports. Encouraging women to utilize antenatal care, interventions such as Sensitization of pregnant women, during ANC visit by the health workers, regarding components of BPCR, danger sign and

Powered by Editorial Manager® and ProduXion Manager® from Aries Systems Corporation

	symptoms during pregnancy, may enhance BPCR.
**Order of Authors:**	Asmita Ghimire, Bachelor
	Bipan Bahadur Tiwari, Bachelor
	Eak Narayan Poudel
	Dr.Mamta Chhetri
	Devraj Regmi
	Laxman Poudel, Bachelor
**Response to Reviewers:**	16 September 2024<br>Subject: Re: Manuscript PONE-D-24-29050R1 – Revisions and Resubmission<br>Dear Dr. Delas Alas Vida,<br>Thank you for your email regarding our manuscript entitled “Birth Preparedness and Complication Readiness Knowledge and Practices Among Recently Delivered Women: A Cross-Sectional Study in Bharatpur, Chitwan, Nepal” (Manuscript ID: PONE-D-24-29050R1).<br>We appreciate your feedback and are addressing the requested revisions as follows:<br>1.Removal of Duplicate Files: We have reviewed the files in our submission and removed all unnecessary or duplicate files. Only the relevant files pertaining to the current version of the manuscript have been included.<br>2.Inclusion of Figure Legends: We have updated the manuscript to include separate, detailed legends for each figure. The legends now provide clear and comprehensive descriptions to enhance the reader's understanding of the figures.<br>3.Reference to Table 9: We have ensured that Table 9 is properly referenced in the text of the manuscript. This reference is included to facilitate easy linking between the text and the table upon acceptance.<br>We have resubmitted the revised manuscript with the requested changes. Please let us know if any further adjustments are needed.<br>Thank you for your consideration and for providing us with the opportunity to revise our manuscript. We look forward to your feedback and hope for a favorable review.<br>Best regards,<br>Asmita Ghimire<br>Chitwan Medical College, Tribhuwan University, Nepal<br>ghimireasmita570@gmail.com<br>+9779866615678
**Additional Information:**	
**Question**	**Response**
**Financial Disclosure**<br><br>Enter a financial disclosure statement that describes the sources of funding for the work included in this submission. Review the submission guidelines for detailed requirements. View published research articles from *PLOS ONE* for specific examples.<br><br>This statement is required for submission and **will appear in the published article** if the submission is accepted. Please make sure it is accurate.	Yes

Powered by Editorial Manager® and ProduXion Manager® from Aries Systems Corporation

**Funded studies**<br>Enter a statement with the following details:<br>• Initials of the authors who received each award<br>• Grant numbers awarded to each author<br>• The full name of each funder<br>• URL of each funder website<br>• Did the sponsors or funders play any role in the study design, data collection and analysis, decision to publish, or preparation of the manuscript?<br><br>Did you receive funding for this work?	
Please add funding details.<br>as follow-up to "**Financial Disclosure**<br><br>Enter a financial disclosure statement that describes the sources of funding for the work included in this submission. Review the submission guidelines for detailed requirements. View published research articles from *PLOS ONE* for specific examples.<br><br>This statement is required for submission and **will appear in the published article** if the submission is accepted. Please make sure it is accurate.<br><br>**Funded studies**<br>Enter a statement with the following details:<br>• Initials of the authors who received each award<br>• Grant numbers awarded to each author<br>• The full name of each funder<br>• URL of each funder website<br>• Did the sponsors or funders play any role in the study design, data collection and analysis, decision to publish, or preparation of the manuscript?<br><br>Did you receive funding for this work?"	Authors name who received awards: Asmita Ghimire<br>Funded by: CMC Research grant Committee<br>URL :https://www.cmc.edu.np/cmc-irc<br><br>The study was funded by Chitwan Medical College Research Grant Committee. However, the funder had no role in study design, data collection and analysis, decision to publish, or preparation of the manuscript.
Please select the country of your main research funder (please select carefully as in some cases this is used in fee calculation).	NEPAL - NP

as follow-up to "**Financial Disclosure**<br><br>Enter a financial disclosure statement that describes the sources of funding for the work included in this submission. Review the submission guidelines for detailed requirements. View published research articles from *PLOS ONE* for specific examples.<br><br>This statement is required for submission and **will appear in the published article** if the submission is accepted. Please make sure it is accurate.<br><br>**Funded studies**<br>Enter a statement with the following details:<br>• Initials of the authors who received each award<br>• Grant numbers awarded to each author<br>• The full name of each funder<br>• URL of each funder website<br>• Did the sponsors or funders play any role in the study design, data collection and analysis, decision to publish, or preparation of the manuscript?<br><br>Did you receive funding for this work?"	
**Competing Interests**<br><br>Use the instructions below to enter a competing interest statement for this submission. On behalf of all authors, disclose any competing interests that could be perceived to bias this work—acknowledging all financial support and any other relevant financial or non-financial competing interests.<br><br>This statement is required for submission and **will appear in the published article** if the submission is accepted. Please make sure it is accurate and that any funding sources listed in your Funding Information later in the submission form are also declared in your Financial Disclosure statement.	The authors have declared that no competing interests exist.

View published research articles from *PLOS ONE* for specific examples.<br><br>**NO authors have competing interests**<br><br>Enter: *The authors have declared that no competing interests exist.*<br><br>**Authors with competing interests**<br><br>Enter competing interest details beginning with this statement:<br><br>*I have read the journal's policy and the authors of this manuscript have the following competing interests: [insert competing interests here]*<br><br>* typeset	
**Ethics Statement**<br><br>Enter an ethics statement for this submission. This statement is required if the study involved:<br><br>• Human participants<br>• Human specimens or tissue<br>• Vertebrate animals or cephalopods<br>• Vertebrate embryos or tissues<br>• Field research<br><br>Write "N/A" if the submission does not require an ethics statement.<br><br>General guidance is provided below. Consult the submission guidelines for detailed instructions. **Make sure that all information entered here is included in the Methods section of the manuscript.**	As my study include Human participants , so before conducting study written informed consent were taken from every participants.<br>Name of Institution review board: Chitwan Medical College-Institution Review Committee<br>CMC-IRC number: CMC-IRC/079/080016<br>Consent: Written

**Format for specific study types**<br><br>**Human Subject Research (involving human participants and/or tissue)**<br>• Give the name of the institutional review board or ethics committee that approved the study<br>• Include the approval number and/or a statement indicating approval of this research<br>• Indicate the form of consent obtained (written/oral) or the reason that consent was not obtained (e.g. the data were analyzed anonymously)<br><br>**Animal Research (involving vertebrate animals, embryos or tissues)**<br>• Provide the name of the Institutional Animal Care and Use Committee (IACUC) or other relevant ethics board that reviewed the study protocol, and indicate whether they approved this research or granted a formal waiver of ethical approval<br>• Include an approval number if one was obtained<br>• If the study involved *non-human primates*, add *additional details* about animal welfare and steps taken to ameliorate suffering<br>• If anesthesia, euthanasia, or any kind of animal sacrifice is part of the study, include briefly which substances and/or methods were applied<br><br>**Field Research**<br><br>Include the following details if this study involves the collection of plant, animal, or other materials from a natural setting:<br>• Field permit number<br>• Name of the institution or relevant body that granted permission	
**Data Availability**<br><br>Authors are required to make all data underlying the findings described fully available, without restriction, and from the time of publication. PLOS allows rare exceptions to address legal and ethical concerns. See the PLOS Data Policy and FAQ for detailed information.	Yes - all data are fully available without restriction

A Data Availability Statement describing where the data can be found is required at submission. Your answers to this question constitute the Data Availability Statement and **will be published in the article**, if accepted.<br><br>**Important:** Stating 'data available on request from the author' is not sufficient. If your data are only available upon request, select 'No' for the first question and explain your exceptional situation in the text box.<br><br>Do the authors confirm that all data underlying the findings described in their manuscript are fully available without restriction?	
**Describe where the data may be found in full sentences. If you are copying our sample text, replace any instances of XXX with the appropriate details.**<br><br>• If the data are **held or will be held in a public repository**, include URLs, accession numbers or DOIs. If this information will only be available after acceptance, indicate this by ticking the box below. For example: *All XXX files are available from the XXX database (accession number(s) XXX, XXX.)*.<br>• If the data are all contained **within the manuscript and/or Supporting Information files**, enter the following: *All relevant data are within the manuscript and its Supporting Information files.*<br>• If neither of these applies but you are able to provide **details of access elsewhere**, with or without limitations, please do so. For example:<br><br>*Data cannot be shared publicly because of [XXX]. Data are available from the XXX Institutional Data Access / Ethics Committee (contact via XXX) for researchers who meet the criteria for access to confidential data.*<br><br>*The data underlying the results presented in the study are available from (include the name of the third party*	The raw data has been made available through given URL below:<br>https://figshare.com/s/3199a871d0bdeae608e3<br>DOI: [10.6084/m9.figshare.26213132]

and contact information or URL).<br>• This text is appropriate if the data are owned by a third party and authors do not have permission to share the data.<br><br>* typeset	
Additional data availability information:	

Powered by Editorial Manager® and ProduXion Manager® from Aries Systems Corporation

Manuscript Click here to access/download;Manuscript;Manuscript.docx

Birth Preparedness and Complication Readiness Knowledge And Practices Among Recently Delivered Women: A Cross-Sectional Study In Bharatpur, Chitwan, Nepal

Asmita Ghimire [1¶*#a], Bipan Bahadur Tiwari[1¶#b], Eak Narayan Poudel[1&], Dr. Mamta Chhetri[1&], Devraj Regmi[1&], Laxman Poudel[2&]

[1] School of Public Health, Chitwan Medical College, Tribhuwan University, Chitwan, Bagmati Nepal;

[2]Health Section, Sitganga Municipality, Arghakhachi, Lumbini, Nepal

[#a]Health Section, Chhatradev Rural Municipality, Arghakhachi, Lumbini, Nepal

[#b]Nepal Development Society, Bharatpur, Chitwan, Bagmati, Nepal

*Corresponding authors

Email:ghimireasmita570@gmail.com

[¶]These authors contributed equally to this work.

[&] These authors also contributed equally to this work.

Abstract

Background

Birth preparedness and complication readiness (BPCR) is a comprehensive strategy, aimed at ensuring that expectant mothers and their families proactively planning for normal delivery and

preparing for potential complications that may arise during pregnancy, labor, delivery, and the postpartum period without any delays.

Objective

This study was conducted to assess birth preparedness and complication readiness knowledge, practices and its associated factors among recently delivered women in Bharatpur city, Chitwan, Nepal.

Methods

A community based cross-sectional study was conducted in 2022, on a sample of 220 recently delivered women. Data were collected using pre-tested structured interview schedule. The collected data were analyzed by IBM SPSS 20 version software. Variables with p-value ≤0.05 on the bivariate analysis were in included in multivariate analysis. Adjusted odds ratios (AOR) with the respective 95% Confidence Interval (CI) and a p-value <0.05 was used to set statistically significant variables in the multivariable analysis.

Results

Among 220 recently delivered women, the majority of the women identified the place of delivery (91.4%) and saved money for childbirth (97.7%). Similarly, most of them arranged transportation (87.3%), identified a companion (85.9%), and arranged necessary materials (90%) for childbirth. Considerable (52.3%) women identified skilled birth attendants. Preparedness for blood donors (36.4%) found to be low as compared to other components. Overall, 46.8% of recently delivered women were well prepared. Family type, knowledge on components of BPCR, obstetric signs and symptoms and ANC visits were found to be statistically significant (p-value<0.05) with birth

preparedness practice. Associated characteristics were further subjected to multivariate logistic regression where knowledge on components of BPCR (AOR=5.34,95%CI: (2.728-10.478) was found significantly associated with BPCR practice.

Conclusion

The overall proportion of women who prepared for birth and its complication readiness was found to be higher as compared to other reports. Encouraging women to utilize antenatal care, interventions such as Sensitization of pregnant women, during ANC visit by the health workers, regarding components of BPCR, danger sign and symptoms during pregnancy, may enhance BPCR.

Introduction

Reducing maternal mortality is a key priority on the international agenda. The new global target is to lower the maternal mortality ratio (MMR) to fewer than 70 maternal deaths per 100,000 live births, with each country aiming to reduce their MMR by at least two-thirds from the 2010 baseline. Additionally, by 2030, no country should have an MMR exceeding 140 deaths per 100,000 live births.[1][2][3]. A safe motherhood strategy namely ‘‘Birth preparedness and complication readiness’’ aims at reducing maternal mortality and morbidity[4], was incorporated in the World Health Organization (WHO) antenatal care package in 2005[5].

Each day, for thousands of women and their families, the event of childbirth becomes a reason of unnecessary suffering due to acute obstetric complications and maternal deaths[6]. United Nation Children Fund Organization (UNICEF) and World Health Organization (WHO) reported that

(585,000) women die annually following complications related to pregnancy and childbirth and 99% of these are in developing countries with about 70% of these mortalities occurring in sub-Saharan Africa[7]. The primary reasons for such mortality were often linked to underutilization of available maternal health services during pregnancy and inadequate preparation for possible complications related to childbirth. [8]. Given the unpredictable nature of childbirth and the associated risk, providing prompt and adequate medical care to women experiencing obstetric complication is vital for reducing these risks. To ensure timely access to skilled maternal and neonatal care during pregnancy, labor, and delivery, it is essential to identify and address any delays. One effective approach to achieve this is through Birth Preparedness and Complication Readiness (BPCR), a core element of globally endorsed safe motherhood initiatives. [9, 10].

Birth Preparedness and Complication Readiness (BP/CR) is a proactive approach that involves planning for a safe, normal birth while being ready to act swiftly in case of emergencies. This comprehensive strategy empowers women, their families, and the community to improve birth planning and respond effectively to emergencies, reducing delays and ensuring better outcomes. [11]. It is estimated that exposure to BPCR interventions results in 18% reduction in neonatal mortality risk and a 28% reduction of in maternal mortality risk [12] So as to reduce the risks associated with pregnancy and childbirth and address factors associated with mortality and morbidity, BPCR strategies have been adopted in Nepal [13] which was rolled out in all 75 districts in 2008-2009 to improve timely access to delivery care services[14].

Birth preparedness motivates people to take proper care during pregnancy and ensure a skilled care provider at every birth. Complication readiness raises awareness of danger signs among women, families and community and prepares them to respond in a proper manner during emergencies[15]. It promotes health care seeking behavior and utilization of appropriate heath care facilities and

skilled personnel for delivery and hence reduce maternal death[16]. In the literature, a host factors were reported as determinants of BPCR such as; knowledge of danger signs of pregnancy, educational level, employment status, adequacy of ANC visits, maternal age, place of residence, poverty and birth order[17].

The goal of reducing maternal mortality and morbidity has fallen woefully behind [18]. According to UN inter-agency, the global maternal mortality ratio declined by 38 per cent – from 342 deaths to 211 deaths per 100,000 live births, in 2000 to 2017 which is less than half to achieve the Sustainable Development global goal[19]. Every day in 2017, approximately 810 women died from preventable causes related to pregnancy and childbirth[20].

Among South Asian regions, India had the highest estimated numbers of maternal deaths, accounting for 12% of global maternal deaths. The proportion of BPCR practices varied from 47% in India, less than a quarter (24.5%) of women was considered well prepared for birth in Bangladesh, 59% in Sri-Lanka, and 32% had not practices any components of BPCR in Nepal [12, 21–23]. Studies from Nepal, Burkina Faso, India, and Nigeria demonstrate that promoting BPCR has increased women's awareness of danger signs and positively influenced their health-seeking behavior during obstetric emergencies. [24–27] . Hence birth preparedness and complication readiness plan is recommended with the notion of pregnancy is risk[28].

In spite of, government and non- government organization focused on to reduce MMR and organized many programmes[29], Nepal is also one of the highest MMR countries in the world with 239 per 100,000 live births in 2016[4]. Approximately (12%) of deaths among women of reproductive age were classified as maternal deaths in 2016, which most could have been prevented through proper practices of BPCR[30]. Only 15% had arranged for transport, (62%) had

saved money, (2%) had identified potential donor, (2%) identify and contact health facilities and health workers while16% had not made any preparations at all[31].

Despite the fact that birth preparedness and complication readiness is essential for further improvement of maternal and child health little is known about the current magnitude and influencing factor in Nepal. To reduce MMR women should prepared for different components of BPCR under Safe motherhood program. This study will be help to access the status of BPCR practice, fill gaps and might be helpful for future researcher and local level authority for planning and implementing BPCR program for women of the community.

Materials and Methods

Study area

Bharatpur is a city in the central-southern part of Nepal. Located in Chitwan district, Bharatpur is the district headquarter of the Chitwan district, as well as a separate Metropolitan authority, and is the fifth largest city of Nepal. There are total of 29 wards and the study was conducted in three wards, 14, 15 and 16 of Bharatpur Metropolitan City. These wards span an area of 12.07, 20.5 and 17.06 square kilometers and comprises, 2094, 1845 and 1894 households respectively, accommodating a total of 33,988 individuals.

Study design

In September 25 to December 29, 2022, a community based cross sectional study was conducted using quantitative methods to assess the knowledge and practices related BPCR**.** The study was

conducted among the recently delivered women who were visiting the three governmental health institutions for immunization of their child within selected wards of Bharatpur, Chitwan during the study period. Those women who were volunteer to participate and respond to the questionnaire were included whereas, exclusion from the study encompassed women who seek vaccination for MR 2nd dose and typhoid, with mental illness, and those unwilling to provide written consent were excluded. This design was chosen because it involves a large participant pool, provides comprehensive details, and generates precise and targeted responses.

Sample Size and Sampling Method

The study's sample size was determined by a hypothesis of 95% confidence interval, with 7% margin of error considering the similar study conducted in similar district with prevalence of poor BPCR practice (29%)[32]. This gave a computed value and the total sample size became 220,

$$n_0 = \frac{\text{z2pq}}{d^2}$$

$$= \frac{1.96^2 * 0.29 * 0.71}{0.06^2}$$

$$= 220$$

Non- Probability convenience sampling technique was used to select study subjects. Firstly, ward no. 14, 15 and 16 was selected purposively as a study site and the total number of governmental health institutions within wards where immunization conducted: health post, Primary health care, out-reach clinic with their respective immunization date were identified. From the above information, three health institution were selected from each ward namely: Shivanagar Primary health care, Fulbari health post and Mangalpur health post along with their out-reach clinic, and

required sample from each health institution was selected conveniently. The data was collected through face-to-face interview after taking written informed consent from the participants.

Measurement

A pre-tested structured interview questionnaire was developed and regularly reviewed in collaboration with the supervisor and subject expert to ensure its alignment with the proposed study objectives. To achieve content validity, items were drawn from the previously validated survey and developed based on an extensive literature search with frequent review to enhance their quality. The questionnaire underwent a pre-testing phase in a different ward of Bharatpur Metropolitan City in the Chitwan district, excluding ward no 14,15 and 16. Necessary adjustments were made accordingly while facilitating accessibility by translating the questionnaire back and forth between Nepali and English languages.

Using a pre tested questionnaire the following information were collected. Socio demographic characteristics including age, ethnicity, religion, education, occupation and average monthly income. The questionnaire included knowledge related questions as knowledge on BPCR components, danger signs and symptoms during pregnancy, labour and postpartum, ANC visits. The women were asked about obstetric factors, institutional factors and role of family and neighbors.

Operational definitions

Level of BPCR Practice: A woman was considered "well prepared" for birth and its complications when she practiced first four critical components among seven basic components **(i)** identified transportation for delivery, ii) saved money, iii) identified health facility, iv) identified

blood donor v) identified skilled health provider, vi) identifying labour and vii) birth companion, preparing necessary materials for mother and baby).[25]

BPCR components knowledge: A woman was considered adequate knowledge on BPCR components when she spontaneously mentioned at least four components.

Knowledge on key danger signs during pregnancy: A woman was considered having adequate knowledge, if she spontaneously at least three danger signs: vaginal bleeding, swollen hands/face, blurred vision, severe abdominal pain, reduced/ no fetal movement, smelling discharge during pregnancy.

Knowledge on key danger signs during labor: A woman was considered having adequate knowledge, if she spontaneously at least three danger signs: severe vaginal bleeding, severe headache, prolonged labor (>12huors), convulsion, and retained placenta during labor/childbirth.

Knowledge on key danger signs during postpartum: A woman was considered having adequate knowledge, if she spontaneously at least three danger signs: severe vaginal bleeding, foul-smelling vaginal discharge, and high fever, unless considered as inadequate knowledge.

Data collection process

The data was collected through face-to-face interview during the immunization date in selected wards institution along with their out-reach clinic which was held from 9-13 of every month, before immunizing the child of respective mother only after getting written informed consent.

Data analysis

The collected data were coded, entered, cleaned and analyzed by IBM SPSS 20 version. Firstly, descriptive statistics, such as frequencies, percentages, and medians were computed to provide an overview of data. Practice level was divided into well prepared and less prepared where, those mothers who followed four critical components among seven were considered “well prepared” and the remaining were considered “less prepared”. Additionally, inferential statistics were employed, specifically the Pearson chi-square test at a significance level of 5%, to explore potential associations between level of practice and selected explanatory variables. A multi-collinearity diagnostic test was conducted to check for collinearity among the independent variables using VIF with the threshold of 10 and there was no multicollinearity detected between the variables.

Data quality control

To maintain the validity of the measurement, questions were drawn from the previously validated survey and developed based on an extensive literature search with frequent review to enhance their quality. The questionnaire underwent a pre-testing phase in other ward of Bharatpur Metropolitan City in the Chitwan district, excluding ward no 14,15 and 16. Necessary adjustments were made accordingly while facilitating accessibility by translating the questionnaire back and forth between local language (Nepali) and English.

Ethical considerations

Ethical approval was obtained from Thesis Committee of the School of Public Health, Chitwan Medical College and Ethical Review Committee of Chitwan Medical College and from Chitwan Medical College Institutional Review Committee (IRC). (Ref: CMC-IRC/079/080-016). Letter of

support was obtained from the health section of Bharatpur Metropolitan Office and respective health institutions before undertaking the study and written informed consent was obtained from the respondents before the interview. For Privacy and confidentiality, all interviews were conducted in private and all cautions were taken to ensure confidentiality. Participants was informed of their rights to deny participation in the study or to withdraw from the study at any stage of data collection. Respondents were provided with the necessary information on importance of birth preparedness and complication readiness to avoid delays in seeking and receiving health care services.

Results

Socio-demographic and obstetric Characteristics

A total of 220 recently delivered women were interviewed. The age range of respondents varied from 17 to 48, with median age of 26. By ethnicity 101(45.9%) were Brahmin/Chhetri and 4(1.8%) were Thakuri/Sanyasi. The major predominant religion includes Hindu 181(82.3%) and Christianity 7(3.2). Occupationally 169(76.8%) were housewives followed by services 10(4.5%). Regarding educational background, 56.6% had secondary education and 12.7% had bachelors and above. The majority of respondents family income 80(36.4%) were between 30-50 thousand. Regarding the birth order, 48.2% of the women had already given birth for one child while 1(0.5%) had four children. About 215(97.7%) of respondents attended antenatal care in their recent pregnancy. Among those women who attended ANC more than four times were 104(47.3%) and 111(50.5%) less than four visits (Table 1).

Table 1: Socio-demographic and obstetric characteristics of recently delivered women, Bharatpur, Chitwan, Nepal (n=220)

Characteristics	Frequency	Percent (%)
**Age**		
17-25	109	49.5
>25	111	50.5
Median/IQR=26/6, Min/Max=17,48		
**Family Type**		
Nuclear	116	52.7
Joint/Extended	104	47.3
**Ethnicity**		
Brahmin/Chhetri	101	45.9
Janajati	66	30
Dalit	42	19.1
Madhesi	7	3.2
Thakuri/Sanyasi	4	1.8
**Religion**		
Hindu	181	82.3

Buddhism	32	15.5
Christianity	7	3.2
**Educational level (n=215)**		
General literate	6	2.7
Basic	56	25.9
Secondary	125	56.6
Bachelors and above	28	12.7
**Mother's occupation**		
Housewife	169	76.8
Agriculture	20	9.1
Business	17	7.7
Services	10	4.5
Labor	2	0.9
Others*	2	0.9
**Monthly income level**		
Less than 10,000	12	5.5
10,000-30,000	88	40
30,000-50,000	80	36.4

More than 50,000	40	18.2
**Birth order**		
One	106	48.2
Two	94	42.7
Three	19	8.6
Four	1	0.5
**Place of delivery**		
Institutional	217	98.6
Home	3	1.4
**ANC visit**		
Yes	215	97.7
No	5	2.3
**Number of ANC (n=215)**		
Less than four times	111	50.5
Greater than or equal to four	104	47.3
**Complication experience**		
Yes	101	45.9
No	119	54.1

Complications (101)		
Smelling discharge	22	22.2
Convulsions	7	7.1
Swollen hands and face	29	29.3
Labor lasting more than 12 hours	7	7.1
Severe headache	13	13.1
Vaginal bleeding	22	22.2
Blurring of vision	2	2
Abdominal pain	38	43.3
Others**	17	17.2

Other*student, other** vomiting, allergy, diabetes, meconium aspiration syndrome, High blood pressure.

Participants Knowledge about key danger signs

Knowledge regarding the components of Birth Preparedness and Complication Readiness (BPCR), 59.1% of women had adequate knowledge. Fig 1 Bar graph depicting the distribution of women's knowledge regarding danger signs associated with pregnancy, childbirth, and the postpartum period. The blue bars represent the percentage of women who demonstrated adequate knowledge by identifying at least three key danger signs: 50.9% for pregnancy, 20.5% for childbirth, and 27.7% for the postpartum period. Conversely, the orange bars indicate the percentage of women with inadequate knowledge, having mentioned fewer than three danger signs: 49.1% for

pregnancy, 79.5% for childbirth, and 72.3% for the postpartum period. When asked about knowledge on ANC checkups, 90.5% of women had knowledge about it while 9.5% did not.

Table 2 outlines the most commonly mentioned items of danger signs during pregnancy were vaginal bleeding 131(65.2%), severe headache 80(39.8%) and swollen hand and face 69(34.3%). During labor and delivery, the majority of listed was severe bleeding 155(86.1), severe headache 68(37.8%) and loss of consciousness 61(33.9%). Similarly, severe bleeding 171(89.1%) was the most commonly mentioned items.

Table 2. Knowledge of respondents on danger signs during pregnancy, labour and post-partum period in Bharatpur, Chitwan, Nepal (n=220)

**Danger sings during pregnancy**	**Frequency**	**Percentage (%)**
Vaginal bleeding	131	65.2
Severe headache	80	39.8
Swollen hand/face	69	34.3
Blurring of vision	36	17.9
Severe lower abdominal pain	134	66.7
Smelling discharge	86	42.8
Convulsion	15	7.5
Reduced/no fetal moment	20	10
**Danger signs during labor/childbirth**		
Severe bleeding	155	86.1
Severe headache	68	37.8

Loss of consciousness	61	33.9
Labor lasting >12 hours	56	31.1
Placenta not delivered 30 min after baby birth	32	17.8
**Danger signs during Postpartum**		
Severe bleeding	171	89.1
Severe headache	72	37.5
Convulsion	45	23.4
High fever	92	47.9
Foul vaginal discharge	24	12.5

Institutional related factors

Table 3 describes the distance to health and delivery facilities, as well as the availability of 24-hour delivery services with median distance to health facilities was 15 minutes, minimum of 2 minutes and a maximum 35 minutes. The majority of participants reported having access to a health facility within 20 minutes. However, over 80% of participants reported a distance of more than 30 minutes to delivery facilities. Additionally, 61.8% of participants reported that 24-hour delivery services were not available. For those whose nearest health facility did not have delivery/24 hours services, 82.3% of respondents reported that their nearest delivery facility was more than 30 minutes away.

Table 3: **Institutional related information among recently delivered women in Bharatpur, Chitwan, Nepal (n=220)**

**Variables**	**Frequency**	**Percentage (%)**
**Distance to health facilities**		
Less than 20 min	168	76.4
More than 20 min	52	23.6
Median/IQR=15/10 MIN, MAX=2, 35		
**Availability of delivery/24hrs facility**		
Yes	84	38.2
No	136	61.8
**Distance to delivery facilities**		
Less than 30 min	39	17.7
More than 30 min	181	82.3

Table 8 presents the support received by the women during delivery from their family and neighbors. The first variable is the decision maker for delivery, which refers to who makes the decision about the place of delivery. The frequency of women who made the decision alone was 43, accounting for 19.5% of the total sample. The frequency of women who made the decision with their husbands was 59, accounting for 26.8% of the total sample. The frequency of women who made the decision jointly with their family members was 105, accounting for 47.7% of the total sample. The frequency of women whose family members alone made the decision was 13, accounting for 5.9% of the total sample.

When asked whether they received assistance from community people, 38.6% of the respondents that they did, while 61.4% did not. For those who did receive assistance, the most common types

of assistance provided were arranging transportation (60.2%) and arranging money (32.5%). Only 3.6% of respondents receiving assistance with arranging blood, while 24.1% reported receiving other types of assistance.

Family and neighbors support related information

Table 4 presents the support received by the women during delivery from their family and neighbors. About 19.5% of women who made the decision alone,26.8% made with their husbands while 5.9% responds that family members alone made decision for delivery. When asked whether they received assistance from community people, 38.6% of the respondents that they did, while 61.4% did not. For those who did receive assistance, the most common types of assistance provided were arranging transportation (60.2%), arranging money (32.5%), arranged blood and 24.1% reported receiving other types of assistance.

Table 4: Support by family and neighbors related information among recently delivered women in Bharatpur, Chitwan, Nepal (n=220)

Variables	Frequency	Percentage (%)
**Decision maker for delivery**		
Women alone	43	19.5
Women and husband	59	26.8
Jointly	105	47.7
Family members alone	13	5.9
**Assistant by community people**		
Yes	85	38.6

No	135	61.4
**If yes, what are those things (n=85)**		
Arrange transportation	50	60.2
Arrange money	27	32.5
Arrange blood	3	3.6
Others*	20	24.1

**denotes multiple response: food, cloths*

Birth Preparedness practices

Fig 2 bar graph illustrating various aspects of childbirth preparedness among women. The orange bars indicate the proportion of women who took specific preparatory actions: 91.4% identified the place of delivery, 97.7% saved money for childbirth, 87.3% arranged transportation, 85.9% selected a companion, and 90% procured necessary materials. In contrast, 52.3% identified skilled birth attendants, and only 36.4% made preparations for blood donors, highlighting a significant gap in blood donor preparedness compared to other aspects of childbirth readiness. (Table 5)

Table 5: Birth preparedness and complication readiness practice among recently delivered women in Bharatpur, Chitwan, Nepal (n=220)

Practices BPCR Components	Frequency	Percentage (%)
**Identified place of delivery**		
Yes	201	91.4
No	19	8.6

**Identified skilled birth attendant**		
Yes	115	52.3
No	105	47.7
**Saved money for childbirth**		
Yes	215	97.7
No	5	2.3
**Arranged Transportation**		
Yes	192	87.3
No	28	12.7
**Identified blood donor**		
Yes	80	36.4
No	140	63.6
**Identified companion**		
Yes	189	85.9
No	31	14.1
**Arranged necessary materials**		
Yes	198	90
No	22	10

Level of BPCR practice

Fig 3 Pie chart depicting the distribution of women's birth preparedness and complication readiness scores. The blue segment represents 103 women (46.8%) who were classified as well-prepared for birth and potential complications, based on key components of birth preparedness and complication readiness. The orange segment represents 117 women (53.2%) who were categorized as less prepared. This distribution highlights the proportion of women with varying levels of preparedness for childbirth and associated complications. (Table 6).

Table 6: Level of Birth Preparedness and Complication Readiness Practices among recently delivered woman in Chitwan, Nepal (n=220)

**Level of BPCR Practiced**	**Frequency**	**Percentage (%)**
Well Prepared	103	46.8
Less Prepared	117	53.2
**Total**	**220**	**100**

Association between level of BPCR Practice and some explanatory variables

In this study, the chi-square test was employed to analyze the relationship between the independent variables and the level of preparedness. The results indicated that significant differences in the level of preparedness were observed concerning family type (P=0.025), knowledge on components of BPCR (P= 0.001), knowledge on signs and symptoms during pregnancy (P=0.002), knowledge on signs and symptoms during labor/childbirth (P-value=0.008), knowledge on signs and symptoms during postpartum (P=0.05) and knowledge on ANC visit as protocol (P= 0.02) (Table

7). However, no significant differences were found in level of preparedness based on age, occupational status, religion, birth order,

Table 7: Association between level of BPCR practices and other explanatory variables among recently delivered woman in Chitwan, Nepal (N=220)

Variables	Level of BPCR practice		Chi-square	p-value
	Well prepared %	Less prepared %		
**Age of Child**				
0-4	47 (50)	47 (50)	3.596	0.166
5-9	21 (36.2)	37 (63.8)		
10-12	35 (51.5)	33 (48.5)		
**Mothers age**				
17-25	46 (42.2)	63 (57.8)	1.849	0.174
>25	57 (51.4)	54 (48.6)		
**Family type**				
Nuclear	46 (39.7)	70(60.3)	5.057	**0.025***
Joint/ Extended	103 (46.8)	47 (45.2)		
**Ethnicity**				
Brahmin/Chhetri	50 (49.5)	51 (50.5)	0.541	0.462
Non-Brahmin/Chhetri	53 (44.5)	66 (55.5)		
**Religion**				
Hindu	84 (46.4)	97 (53.6)	0.069	0.793
Non-Hindu	19 (48.7)	20 (51.3)		

**Mother education (n=215)**				
General/basic	27 (43.5)	35 (56.5)	0.663	0.415
Secondary and above	76 (49.7)	77 (50.3)		
**Mother occupation**				
Housewife/agriculture	85 (45)	104 (55)	1.833	0.176
Other than housewife	18 (58.1)	13 (41.9)		
**Monthly income**				
Less than 50,000	41 (41)	59 (59)	2.493	0.114
More than 50,000	62 (51.7)	58 (48.3)		
**Age at marriage**				
Teenage (≤19)	36 (45)	44 (55)	0.167	0.683
Above teenage (>19)	67 (47.9)	73 (52.1)		
**Knowledge on components of BPCR**				
Adequate	83 (63.8)	47 (36.2)	37.00	**<0.001***
Inadequate	20 (22.2)	70 (77.8)		
**Knowledge on signs and symptoms during pregnancy**				
Adequate	64 (57.1)	48 (42.9)	9.768	**0.002***
Inadequate	39 (36.1)	69 (63.9)		
**Knowledge on signs and**				

**symptoms during childbirth**				
Adequate	29 (57.1)	16 (42.9)	7.059	**0.008***
Inadequate	74 (36.1)	101 (63.9)		
**Knowledge on signs and symptoms during postpartum**				
Adequate	35 (57.4)	26 (42.6)	3.779	**0.05***
Inadequate	68 (42.8)	91 (57.2)		
**Knowledge on ANC visit protocol**				
Yes	100 (50.3)	99 (49.7)	9.868	**0.02***
No	3 (14.3)	18 (85.7)		
**Times of child bear**				
≤2	91 (45.5)	109 (54.5)	1.535	0.215
>2	12 (60)	8(40)		
**ANC visit**				
Yes	103 (47.9)	112 (52.1)		0.062^{β}
No	0 (0)	5 (100)		
**Number of ANC visit**				
Less than four times	6 (24)	19 (76)	5.535	**0.019***
Greater than or equal to four times	93 (48.9)	97 (51.1)		

**Place of delivery**				
Home	0 (0)	3 (1.4)		0.250 $^{\beta}$
Institutional	103 (47.5)	114 (51.8)		
**Complication experiences**				
Yes	48 (47.5)	53 (52.8)	0.037	0.847
No	55 (46.2)	64 (53.8)		

**Denotes significant association (By applying Pearson chi-square test at 5% level of significance) and β denotes association by applying Fisher's Exact test at 5% level of significance.*

Test of multicollinearity between independent variables and Birth preparedness and complication readiness (BPCR)

Table 8 presents that, those independent variables which where statistically significant and associated with birth preparedness and complication readiness practices were further used to test the multicollinearity among those independent variables.

Table 8: Test of multicollinearity between independent variables and BPCR practice among recently delivered woman in Chitwan, Nepal (n=220)

**Independent variables**	**Tolerance**	**VIF (Variance Inflation Factor**
Family type	0.978	1.023
Knowledge on components of BPCR	0.816	1.226

Knowledge on key danger signs during pregnancy	0.715	1.399
Knowledge on key danger signs during labor	0.611	1.636
Knowledge on key danger signs during post-partum	0.584	1.711
Number of ANC visits	0.930	1.075

Multivariate analysis of factors associated with BPCR practice

Six characteristics that exhibit statistically significant association (p<0.05) with birth preparedness and complication readiness practice at 95% CI during bivariate analysis were further subjected to multivariate logistic regression for adjustment of possible confounder. Multicollinearity test was done. None of them have tolerance <0.1 and VIF >10. There was no problem of serious collinearity among independent variables.

Birth preparedness practice was found to be statistically significant (p<0.05) with knowledge on components of BPCR. Women with adequate knowledge on components of BPCR are 5 times more likely to practice or well prepared for birth and complication as compared to women with inadequate knowledge (AOR=5.34,95%CI: (2.728-10.478). Table 9

Table 9: Multivariate analysis of factors associated with BPCR practice among recently delivered woman in Chitwan, Nepal (n=220)

**Variable**	**COR (95% CI)**	**AOR (95%CI)**	**p-value**
**Family Type**			
Nuclear	*Ref*	*Ref*	0.88
Joint/Extended	1.846(1.080-3.155)	1.689(0.926-3.080)	
**Knowledge on components of BPCR**			
Inadequate	*Ref*	*Ref*	**0.00**
Adequate	6.181(3.351-11.402)	**5.347(2.728-10.478) ***	
**Knowledge on key danger signs during pregnancy**			
Inadequate	*Ref*	*Ref*	
Adequate	2.359(1.371-4.058)	1.332(0.669-2.652)	0.414
**Knowledge on key danger signs during labor**			
Inadequate	*Ref*	*Ref*	0.675
Adequate	2.474(1.253-4.883)	1.213(0.492-2.991)	
**Knowledge on key danger signs during postpartum**			
Inadequate	*Ref*	*Ref*	0.605
Adequate	1.801(0.992-3.272)	0.801(0.346-1.856)	
**Knowledge on ANC visit as**			
Less than four times	*Ref*	*Ref*	0.14
Greater than four times	2.499(1.451-4.305)	2.151(1.170-3.954)	

*Variables significant at $p<0.05$ *Ref*: Reference Category CI: Confidence Interval OR: Odds Ratio

Discussions

BPCR is one of the proven and effective health care strategy in preventing maternal mortality especially for countries with prevailing high risk of maternal deaths and inefficient health care system. The study found that the overall prevalence of BPCR was 46.8%, which is comparable to rates reported in studies conducted in Southern Nigeria (48.4%)[33] and Southern Ethiopia (48.5%)[10], Sidama (17%), Wolyita (18.3%) zones of Ethiopia[34, 35]. However, the prevalence rate was better than that reported in a study conducted in Dang district (44.36%)[21]. On the other hand, the rate was lower than rates reported in studies conducted in Rapti municipality, Kaski district[20, 36] and other South Asian countries, including Sri Lanka, Karnataka, India, and West Bengal[37, 38], as well as Southwest Nigeria and Tanzania[16, 39]. The most common birth preparedness practice observed in this study was saving money, which may be explained by the fact that both women and their partners know that money is required to facilitate referral in case of complications. And it is interesting to note that other studies on birth preparedness have reported similar findings[40, 41]. The study found that identification of blood donor as an obstetric emergency was not considered crucial issue by some respondents as other components where only 34% identified potential blood donor. However, other study reported even lower percent of respondents practiced this component[9]. The variation in findings may be attributed to several factors, such as differences in the study population, with our study involving recently delivered women, while the studies in South Ethiopia[34] and Dang[42] included pregnant women and both

delivered and pregnant women, respectively. Pregnant women may be unable to report their preparedness for situations they have not yet experienced. Additionally, variations in female literacy levels, empowerment, study setting, socio-cultural difference, and methodological differences in assessing BPCR could also contribute to the differences in findings.

Nearly three-quarters (72.1%) and over one-third (45.3%) of the study were knowledgeable about identifying place of delivery and arranging transportation as essential components of BPCR, this aligns with similar findings reported in other studies. In a survey conducted in Samara Logia Town, where 75% and 45% of participants were knowledgeable on those components[43]. The study revealed that 59.1%, 50.9%, of participants had knowledge about the components of BPCR and danger signs during pregnancy This finding is in contrast to a study conducted in Farta district, Ethiopia, which reported a significantly lower percentage of 14.3% and 23.3% of participants with knowledge of the components and danger signs during pregnancy respectively[11]. The study revealed that the participants' understanding of danger signs during labor/childbirth and the postpartum period was inadequate, with only 20.5% and 27.3% of them reporting familiarity with these signs, respectively which is consistent with the study done in Sodo town, Southern Ethiopia,[10]The finding that severe bleeding was identified as a major sign and symptom during childbirth is similar in both the current study and a study conducted in Mizan-Aman, Southwest Ethiopia[39]. Women having knowledge of obstetric danger signs might have chance to improve their decision-making ability on their maternal and child health care service utilization and can respond immediately in the event of emergencies to avoid delays[11].

Approximately Ninety Eight Percent of the women have had at least one ANC visit, about half (47.3%) attended the minimum recommended number of four or more visits, was found to be low as compared to the national NDHS data (81%)[44] and studies in Kassena- Nankana of Ghana[45].

This study included a specific decision-making variable which is absent from most studies. But in this study almost half of them made decision jointly regarding the place of delivery as in the study in Rapti municipality[32].

Our study identified that the level of BPCR practice is significantly associated with family type (χ^2=5.057, p=0.025)and knowledge on ANC visit as protocol (χ^2=9.868, p=0.02) which is compliance with the findings done in Kenya and Tanzania and Rapti Municipality of Chitwan, Nepal[32, 46]. Similarly, knowledge on components of BPCR ((χ^2=37, p=0.001), danger sign and symptoms during pregnancy (χ^2=9.768, p=0.002), labor/childbirth and postpartum (χ^2=3.779, p=0.05) are significantly associated with BPCR practice, which is similar with the findings of community- based study done in rural area in Bangladesh, Mizan-Aman Town, Southwest Ethiopia and Nepal where association between knowledge and practice was observed[6, 22]. Also, the current study found that the BPCR practice was statistically significant with number of ANC visits. This finding is supported by the study in central Tanzania where woman who had attended 4 or more times antenatal care were better prepared than women who had attended <4 times antenatal care[47]. The significant influence of woman's literacy level on BPCR practice is highlighted by many studies from Sri-lanka and African countries[48]. However, in our study and a study in Biratnagar, Nepal, Karnataka India, Bangladesh, woman's education level did not show a significant association with BPCR[15, 40, 49]. This could be attributed to overall high (97.7%) literacy rate in this study population.

Having the knowledge of BPCR increases the practice of BPCR among recently delivered women and 5 times more likely to be well prepared according to this study. This is comparable with the studies from Central Ethiopia [50] Bench Maji zone [51] and Farta district [11]. The inference of

this finding could be once women become knowledgeable about BPCR, they are expected to practice the components of BPCR and ready to act upon it when it occurs.

The advantage of asking women who are recently delivered about the birth preparedness as they have completed their pregnancies and already made all necessary arrangement. As there may be variation in their service use in pregnant women, they will not provide the information based on their plan to use those services.

Conclusions

Although not satisfactory in the view of expectations, a relatively higher practice of BPCR had been observed in the study area compared to other reports. Majority of participants saved money as one of preparation for childbirth while only few prepared blood donors in case of obstetric complication and emergency. This study also revealed that the respondent's knowledge of danger sign and symptoms during pregnancy was adequate but during child birth and postpartum was low. Similarly, the women having recommended ANC visit was low as compared to national data. These findings lend support to reinforce health education on BPACR during pregnancy. Further the principal factors affecting the BPCR practice were family type, ANC visit, knowledge on components of BPCR, danger signs during pregnancy, labor/Childbirth, and postpartum period, which suggests more intervention to be taken at health institution and health care providers. Furthermore, pregnant women should continuously be encouraged to attend ANC as per the protocol so that interventions such as Sensitization of pregnant women, during ANC visit by the health workers, regarding Components of BPCR, danger sign and symptoms during pregnancy, may enhance BPCR.

Study Limitations

- Self-reported practice may compromise the validity of the findings.
- There could also be information bias as the questions were asked to all those who delivered in the last 12 months. Some might not have recalled the danger signs, and symptoms.

Acknowledgements

We would like to thank school of public health, Chitwan Medical college, health section of Bharatpur metropolitan city and health institutions for providing support in undertaking this research.

S1 File. SPSS dataset BPCR.

https://figshare.com/s/3199a871d0bdeae608e3

DOI: [10.6084/m9.figshare.26213132]

References

[1] Nations U. Targets and Strategies for Ending Preventable Maternal Mortality consensus statement.

[2] Mgawadere F, Kana T, Van Den Broek N. Measuring maternal mortality: A systematic review of methods used to obtain estimates of the Maternal Mortality Ratio (MMR) in low- and middle-income countries. *Br Med Bull* 2017; 121: 121–134.

[3] United Nations . Sustainable Development Goals. GenevaWorld Health Organization, 2015., http://www.un.org/sustainabledevelopment/sustainable-development-goals/.

[4] Basnet I. National Safe Motherhood Plan (2002-2017). 2002; 1–23.

[5] Ihomba PK, Nyamari JM, Were T. Birth Preparedness and Complication Readiness among Women with Preg-. *East African Heal Res J* 2020; 4: 26–32.

[6] Moinuddin M, Christou A, Hoque DME, et al. Birth preparedness and complication readiness (BPCR) among pregnant women in hard-to-reach areas in Bangladesh. *PLoS One* 2017; 12: 1–19.

[7] Yılmaz S, Calikoglu EO, Kosan Z. Several studies across the world suggest that promoting BPACR interventions improves preventive behaviours, improves knowledge of mothers about danger signs and leads to improvement in care- seeking during obstetric emergency. *Niger J Clin Pract* 2019; 22: 1070–1077.

[8] Zepre K, Kaba M. Birth preparedness and complication readiness among rural women of reproductive age in Aabeshige district, Gguraghe zone, SNNPR, Eethiopia. *Int J Womens Health* 2017; 9: 11–21.

[9] Affipunguh PK, Laar AS. Assessment of knowledge and practice towards birth preparedness and complication readiness among women in Northern Ghana : a cross-sectional study. 2016; 2: 121–129.

[10] Azeze GA, Mokonnon TM, Kercho MW. Birth preparedness and complication readiness practice and influencing factors among women in Sodo town, Wolaita zone, Southern Ethiopia, 2018; Community based cross-sectional study. *Reprod Health*; 16. Epub ahead of print 29 March 2019. DOI: 10.1186/s12978-019-0703-z.

[11] Limenih MA, Belay HG, Tassew HA. Birth preparedness , readiness planning and associated factors among mothers in Farta district , Ethiopia : a cross-sectional study. 2019; 4: 1–10.

[12] Wudu MA, Tsegaye TB. Birth preparedness and complication readiness and associated factors among recently delivered mothers in mizan-aman town, Southwest Ethiopia, 2019. *Int J Womens Health* 2021; 13: 177–187.

[13] Pervin J, Tin Nu U, Rahman AMQ, et al. Level and determinants of birth preparedness and complication readiness among pregnant women: A cross sectional study in a rural area in Bangladesh. *PLoS One*; 13. Epub ahead of print 1 December 2018. DOI: 10.1371/journal.pone.0209076.

[14] Danna VA. Utility of the three-delays model and its potential for supporting a solution-based approach to accessing intrapartum care in low- and middle-income countries. A qualitative evidence synthesis.

[15] Moran AC, Sangli G, Dineen R, et al. Birth-preparedness for maternal health: Findings from Koupéla district, Burkina Faso. *J Heal Popul Nutr* 2006; 24: 489–497.

[16] Soubeiga D, Gauvin L, Hatem MA, et al. Birth Preparedness and Complication Readiness (BPCR) interventions to reduce maternal and neonatal mortality in developing countries : systematic review and meta-analysis. 2014; 1–11.

[17] Safe Motherhood Program in Nepal Evolution and Impact Public health in Nepal. Blogspot.com accessed at August 6 mondey.

[18] UNFPA Asiapacific | Maternal health, https://asiapacific.unfpa.org/en/maternal-health (accessed 5 August 2022).

[19] Sitaula S, Basnet T, Agrawal A, et al. Prevalence and risk factors for maternal mortality at a tertiary care centre in Eastern Nepal- retrospective cross sectional study. *BMC Pregnancy Childbirth* 2021; 21: 1–8.

[20] Marta Levitt-Dayal. Saving Mothers' Lives: What works, a field guide for implementing best practices in safe motherhood. 2002; 32–56.

[21] Ekabua JE, Ekabua KJ, Odusolu P, et al. Awareness of Birth Preparedness and Complication Readiness in Southeastern Nigeria. 2011. Epub ahead of print 2011. DOI: 10.5402/2011/560641.

[22] South Asia's Quest for Reduced Maternal Mortality: What the Data Show, https://blogs.worldbank.org/health/south-asia-s-quest-reduced-maternal-mortality-what-data-show (accessed 5 August 2022).

[23] Nadeeka Perera SEG. Birth and emergency preparedness and associated factors among postnatal mothers at Base Hospital Balangoda, Sri Lanka.

[24] Ministry of Health N, 2017. l; NE and I. Nepal Demographic and Health Survey 2016.

Kathmandu, Nepal: Ministry of Health, Nepal. *Eugen Q* 1967; 14: 54–59.

[25] Mukhopadhyay DK, Mukhopadhyay S, Bhattacharjee S, et al. Status of birth preparedness and complication readiness in Uttar Dinajpur District, West Bengal. *Indian J Public Health* 2013; 57: 147–154.

[26] Bintabara D, Mohamed MA, Mghamba J, et al. Birth preparedness and complication readiness among recently delivered women in chamwino district, central Tanzania: A cross sectional study 'Obstetrics'. *Reprod Health*; 12. Epub ahead of print 16 May 2015. DOI: 10.1186/s12978-015-0041-8.

[27] 10057-Article Text-51571-1-10-20080827.

[28] Osaze D, Efegbere HA, Akwa OP. Correlates of birth preparedness practices and complication readiness among women in eight communities in Delta State, Nigeria Daniel. 2021; 244–252.

[29] Saaka M, Alhassan L. Prevalence and predictors of birth preparedness and complication readiness in the Kassena-Nankana district of Ghana: An analytical cross-sectional study. *BMJ Open* 2021; 11: 1–9.

[30] Bal Kumar KC. Ministry of Health and Population (MOHP) [Nepal], New ERA, and ICF International Inc. 2012. Nepal Demographic and Health Survey 2011. Kathmandu, Nepal: Ministry of Health and Population, New ERA, and ICF International, Calverton, Maryland. *Asian Pacific Migr J* 2008; 17: 287–309.

[31] NSO. *National Population and Housing Census 2021*. 2021.

[32] Silwal K, Poudyal JK, Shah R, et al. Factors Influencing Birth Preparedness in Rapti

Municipality of Chitwan, Nepal. *Int J Pediatr (United Kingdom)*; 2020. Epub ahead of print 2020. DOI: 10.1155/2020/7402163.

[33] Mcpherson RA, Khadka N, Moore JM, et al. Are Birth-preparedness Programmes Effective? Results From a Field Trial in Siraha District, Nepal. *J Heal Popul NUTR* 2006; 24: 479–488.

[34] Hailu M, Gebremariam A, Alemseged F, et al. Birth preparedness and complication readiness among pregnant women in Southern Ethiopia. *PLoS One*; 6. Epub ahead of print 2011. DOI: 10.1371/journal.pone.0021432.

[35] Gebre M, Gebremariam A, Abebe TA. Birth preparedness and complication readiness among pregnant women in Duguna Fango District, Wolayta Zone, Ethiopia. *PLoS One* 2015; 10: 1–12.

[36] Health J, Nutr P. Birth Preparedness and Complication Readiness among Slum Women in Indore City, India. 2010; 28: 383–391.

[37] Trends in maternal mortality: 2000 to 2017: estimates by WHO, UNICEF, UNFPA, World Bank Group and the United Nations Population Division. Geneva: World Health Organization; 2019.

[38] Health topics, https://www.who.int/health-topics/#M (accessed 4 August 2022).

[39] Mahadevia G, Vikas M. International Institute for Population Sciences (IIPS) and ICF. 2017. National Family Health Survey (NFHS-4), 2015-16: India. Mumbai: IIPS. *Int Sci J Environ Sci* 2012; 21: 7–15.

[40] Akshaya KM, Shivalli S. Birth preparedness and complication readiness among the

women beneficiaries of selected rural primary health centers of Dakshina Kannada district, Karnataka, India. *PLoS One*; 12. Epub ahead of print 1 August 2017. DOI: 10.1371/journal.pone.0183739.

[41] Afik A. The relationship between response time and patient survival with emergency treatment by the Code Blue Team. *Indian J Public Heal Res Dev* 2018; 9: 1803–1807.

[42] Bhusal CK, Bhattarai S. INVOLVEMENT OF MALE IN BIRTH PREPAREDNESS IN TULSIPUR MUNICIPALITY OF DANG DISTRICT NEPAL, www.jcmc.cmc.edu.np (2015).

[43] Estimates by WHO, UNICEF, UNFPA WBG and the UNPD. TRENDS IN MATERNAL MORTALITY2000 to 2017.

[44] Debelew GT, Afework F, Yalew AW. Factors affecting birth preparedness and complication readiness in Jimma Zone, Southwest Ethiopia: A multilevel analysis. *Pan Afr Med J*; 19. Epub ahead of print 11 November 2014. DOI: 10.11604/pamj.2014.19.272.4244.

[45] Florence M, Atuhaire C, Nkfusai CN, et al. Knowledge and practice of birth preparedness and complication readiness among pregnant women attending antenatal clinic in openzinzi hciii, adjumani district, Uganda. *Pan Afr Med J* 2019; 34: 1–9.

[46] Orwa J, Gatimu SM, Mantel M, et al. Birth preparedness and complication readiness among women of reproductive age in Kenya and Tanzania: a community-based cross-sectional survey. *BMC Pregnancy Childbirth*; 20. Epub ahead of print 1 December 2020. DOI: 10.1186/s12884-020-03329-5.

[47] Ibadin SH, Adam VY, Adeleye OA, et al. Birth preparedness and complication readiness among pregnant women in a rural community in Southern Nigeria. *S Afr J Obstet Gynaecol* 2016; 22: 47–51.

[48] Rodrigo CH, Kumarapeli V. Birth preparedness, complication readiness and associated factors among pregnant women seeking antenatal care at a Medical Officer of Health (MOH) area in Sri Lanka. *J Postgrad Inst Med* 2019; 6: 84.

[49] Subedi S. Knowledge and Practice on Birth Preparedness and Complication Readiness among Pregnant Women in Selected Ward of Biratnager Municipality, Nepal. *Tribhuvan Univ J* 2019; 33: 53–66.

[50] Girma D, Waleligne A, Dejene H. Birth preparedness and complication readiness practice and associated factors among pregnant women in Central Ethiopia, 2021: A cross-sectional study. *PLoS One* 2022; 17: 1–16.

[51] Gudeta TA, Regassa TM. Factors Associated with Birth Preparedness and Complication Readiness Among Pregnant Women in Bench Maji Zone, Southwest Ethiopia: A Community-Based Cross-Sectional Study. *Ethiop J Health Sci* 2019; 29: 567–576.

Fig 2: Practices of BPCR Components among recently delivered women in Bharatpur, Nepal (n=220)

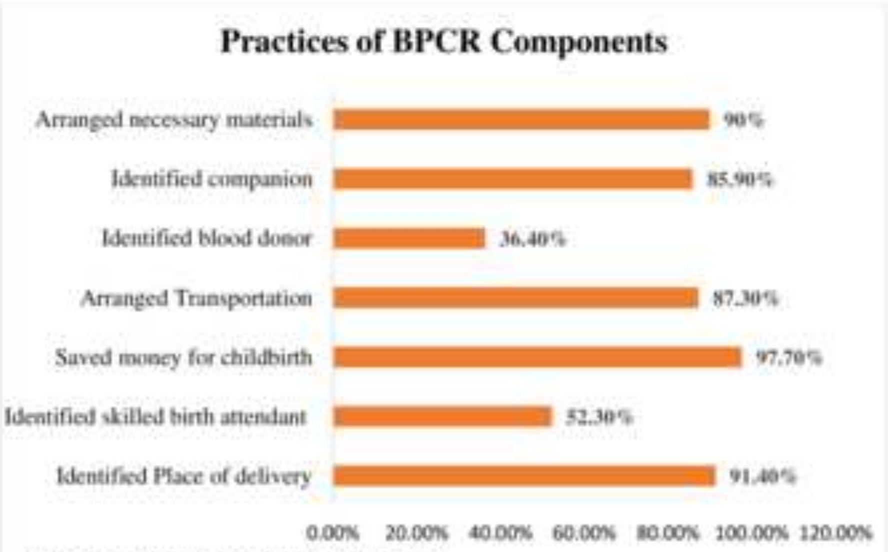

Fig 2: Practices of BPCR Components

 Click here to access/download;Figure;Fig 3 manuscript.tiff

Fig 3: Level of BPCR Practices among recently delivered women in Bharatpur, Nepal (n=220)

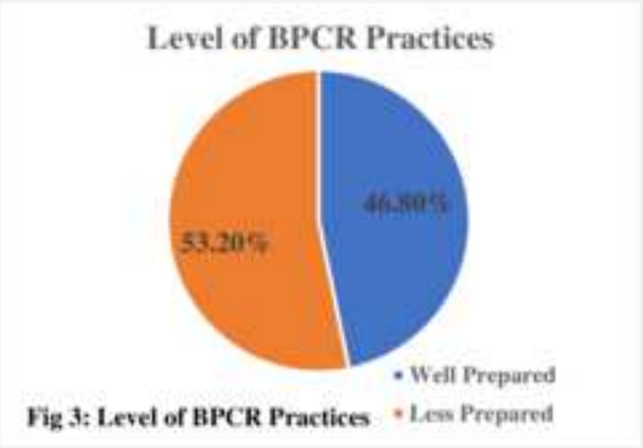

Fig 3: Level of BPCR Practices

Figure

Click here to access/download;Figure;Fig-1-Manuscript.tiff

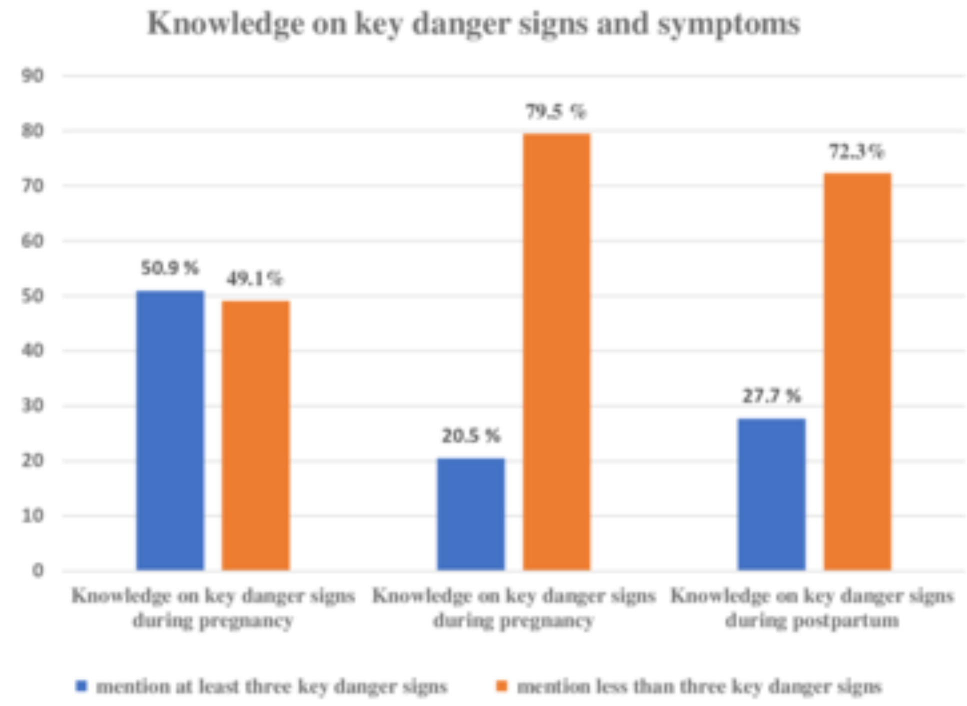

Fig 1: Knowledge on Key danger signs and symptoms among recently delivered women in Bharatpur, Nepal (n=220)

Click here to access/download
Supporting Information
FInal data 101.sav

Birth Preparedness and Complication Readiness Knowledge And Practices Among Recently Delivered Women: A Cross-Sectional Study In Bharatpur, Chitwan, Nepal

Asmita Ghimire [1¶*#a], Bipan Bahadur Tiwari[1¶#b], Eak Narayan Poudel[1&], Dr. Mamta Chhetri[1&], Devraj Regmi[1&], Laxman Poudel[2&]

[1] School of Public Health, Chitwan Medical College, Tribhuwan University, Chitwan, Bagmati Nepal;

[2]Health Section, Sitganga Municipality, Arghakhachi, Lumbini, Nepal

[#a]Health Section, Chhatradev Rural Municipality, Arghakhachi, Lumbini, Nepal

[#b]Nepal Development Society, Bharatpur, Chitwan, Bagmati, Nepal

*Corresponding authors

Email:ghimireasmita570@gmail.com

[¶]These authors contributed equally to this work.

[&] These authors also contributed equally to this work.

Abstract

Background

Birth preparedness and complication readiness (BPCR) is a comprehensive strategy, aimed at ensuring that expectant mothers and their families proactively planning for normal delivery and preparing for potential complications that may arise during pregnancy, labor, delivery, and the postpartum period without any delays.

Objective

This study was conducted to assess birth preparedness and complication readiness knowledge, practices and its associated factors among recently delivered women in Bharatpur city, Chitwan, Nepal.

Methods

A community based cross-sectional study was conducted in 2022, on a sample of 220 recently delivered women. Data were collected using pre-tested structured interview schedule. The collected data were analyzed by IBM SPSS 20 version software. Variables with p-value ≤ 0.05 on the bivariate analysis were in included in multivariate analysis. Adjusted odds ratios (AOR) with the respective 95% Confidence Interval (CI) and a p-value <0.05 was used to set statistically significant variables in the multivariable analysis.

Results

Among 220 recently delivered women, the majority of the women identified the place of delivery (91.4%) and saved money for childbirth (97.7%). Similarly, most of them arranged transportation (87.3%), identified a companion (85.9%), and arranged necessary materials (90%) for childbirth. Considerable (52.3%) women identified skilled birth attendants. Preparedness for blood donors (36.4%) found to be low as compared to other components. Overall, 46.8% of recently delivered

women were well prepared. Family type, knowledge on components of BPCR, obstetric signs and symptoms and ANC visits were found to be statistically significant (p-value <0.05) with birth preparedness practice. Associated characteristics were further subjected to multivariate logistic regression where knowledge on components of BPCR (AOR=5.34,95%CI: (2.728-10.478) was found significantly associated with BPCR practice.

Conclusion

The overall proportion of women who prepared for birth and its complication readiness was found to be higher as compared to other reports. Encouraging women to utilize antenatal care, interventions such as Sensitization of pregnant women, during ANC visit by the health workers, regarding components of BPCR, danger sign and symptoms during pregnancy, may enhance BPCR.

Introduction

Reducing maternal mortality is a key priority on the international agenda. The new global target is to lower the maternal mortality ratio (MMR) to fewer than 70 maternal deaths per 100,000 live births, with each country aiming to reduce their MMR by at least two-thirds from the 2010 baseline. Additionally, by 2030, no country should have an MMR exceeding 140 deaths per 100,000 live births.[1][2][3]. A safe motherhood strategy namely ‘‘Birth preparedness and complication readiness’’ aims at reducing maternal mortality and morbidity[4], was incorporated in the World Health Organization (WHO) antenatal care package in 2005[5].

Each day, for thousands of women and their families, the event of childbirth becomes a reason of unnecessary suffering due to acute obstetric complications and maternal deaths[6]. United Nation Children Fund Organization (UNICEF) and World Health Organization (WHO) reported that (585,000) women die annually following complications related to pregnancy and childbirth and 99% of these are in developing countries with about 70% of these mortalities occurring in sub-Saharan Africa[7]. The primary reasons for such mortality were often linked to underutilization of available maternal health services during pregnancy and inadequate preparation for possible complications related to childbirth. [8]. Given the unpredictable nature of childbirth and the associated risk, providing prompt and adequate medical care to women experiencing obstetric complication is vital for reducing these risks. To ensure timely access to skilled maternal and neonatal care during pregnancy, labor, and delivery, it is essential to identify and address any delays. One effective approach to achieve this is through Birth Preparedness and Complication Readiness (BPCR), a core element of globally endorsed safe motherhood initiatives. [9, 10].

Birth Preparedness and Complication Readiness (BP/CR) is a proactive approach that involves planning for a safe, normal birth while being ready to act swiftly in case of emergencies. This comprehensive strategy empowers women, their families, and the community to improve birth planning and respond effectively to emergencies, reducing delays and ensuring better outcomes. [11]. It is estimated that exposure to BPCR interventions results in 18% reduction in neonatal mortality risk and a 28% reduction of in maternal mortality risk [12] So as to reduce the risks associated with pregnancy and childbirth and address factors associated with mortality and morbidity, BPCR strategies have been adopted in Nepal [13] which was rolled out in all 75 districts in 2008-2009 to improve timely access to delivery care services[14].

Birth preparedness motivates people to take proper care during pregnancy and ensure a skilled care provider at every birth. Complication readiness raises awareness of danger signs among women, families and community and prepares them to respond in a proper manner during emergencies[15]. It promotes health care seeking behavior and utilization of appropriate heath care facilities and skilled personnel for delivery and hence reduce maternal death[16]. In the literature, a host factors were reported as determinants of BPCR such as; knowledge of danger signs of pregnancy, educational level, employment status, adequacy of ANC visits, maternal age, place of residence, poverty and birth order[17].

The goal of reducing maternal mortality and morbidity has fallen woefully behind [18]. According to UN inter-agency, the global maternal mortality ratio declined by 38 per cent – from 342 deaths to 211 deaths per 100,000 live births, in 2000 to 2017 which is less than half to achieve the Sustainable Development global goal[19]. Every day in 2017, approximately 810 women died from preventable causes related to pregnancy and childbirth[20].

Among South Asian regions, India had the highest estimated numbers of maternal deaths, accounting for 12% of global maternal deaths. The proportion of BPCR practices varied from 47% in India, less than a quarter (24.5%) of women was considered well prepared for birth in Bangladesh, 59% in Sri-Lanka, and 32% had not practices any components of BPCR in Nepal [12, 21–23]. Studies from Nepal, Burkina Faso, India, and Nigeria demonstrate that promoting BPCR has increased women's awareness of danger signs and positively influenced their health-seeking behavior during obstetric emergencies. [24–27] . Hence birth preparedness and complication readiness plan is recommended with the notion of pregnancy is risk[28].

In spite of, government and non- government organization focused on to reduce MMR and organized many programmes[29], Nepal is also one of the highest MMR countries in the world with 239 per 100,000 live births in 2016[4]. Approximately (12%) of deaths among women of reproductive age were classified as maternal deaths in 2016, which most could have been prevented through proper practices of BPCR[30]. Only 15% had arranged for transport, (62%) had saved money, (2%) had identified potential donor, (2%) identify and contact health facilities and health workers while16% had not made any preparations at all[31].

Despite the fact that birth preparedness and complication readiness is essential for further improvement of maternal and child health little is known about the current magnitude and influencing factor in Nepal. To reduce MMR women should prepared for different components of BPCR under Safe motherhood program. This study will be help to access the status of BPCR practice, fill gaps and might be helpful for future researcher and local level authority for planning and implementing BPCR program for women of the community.

Materials and Methods

Study area

Bharatpur is a city in the central-southern part of Nepal. Located in Chitwan district, Bharatpur is the district headquarter of the Chitwan district, as well as a separate Metropolitan authority, and is the fifth largest city of Nepal. There are total of 29 wards and the study was conducted in three wards, 14, 15 and 16 of Bharatpur Metropolitan City. These wards span an area of 12.07, 20.5 and

17.06 square kilometers and comprises, 2094, 1845 and 1894 households respectively, accommodating a total of 33,988 individuals.

Study design

In September 25 to December 29, 2022, a community based cross sectional study was conducted using quantitative methods to assess the knowledge and practices related BPCR**.** The study was conducted among the recently delivered women who were visiting the three governmental health institutions for immunization of their child within selected wards of Bharatpur, Chitwan during the study period. Those women who were volunteer to participate and respond to the questionnaire were included whereas, exclusion from the study encompassed women who seek vaccination for MR 2nd dose and typhoid, with mental illness, and those unwilling to provide written consent were excluded. This design was chosen because it involves a large participant pool, provides comprehensive details, and generates precise and targeted responses.

Sample Size and Sampling Method

The study's sample size was determined by a hypothesis of 95% confidence interval, with 7% margin of error considering the similar study conducted in similar district with prevalence of poor BPCR practice (29%)[32]. This gave a computed value and the total sample size became 220,

$$n_0 = \frac{\text{z2pq}}{d^2}$$

$$= \frac{1.96^2 * 0.29 * 0.71}{0.06^2}$$

$$= 220$$

Non- Probability convenience sampling technique was used to select study subjects. Firstly, ward no. 14, 15 and 16 was selected purposively as a study site and the total number of governmental health institutions within wards where immunization conducted: health post, Primary health care, out-reach clinic with their respective immunization date were identified. From the above information, three health institution were selected from each ward namely: Shivanagar Primary health care, Fulbari health post and Mangalpur health post along with their out-reach clinic, and required sample from each health institution was selected conveniently. The data was collected through face-to-face interview after taking written informed consent from the participants.

Measurement

A pre-tested structured interview questionnaire was developed and regularly reviewed in collaboration with the supervisor and subject expert to ensure its alignment with the proposed study objectives. To achieve content validity, items were drawn from the previously validated survey and developed based on an extensive literature search with frequent review to enhance their quality. The questionnaire underwent a pre-testing phase in a different ward of Bharatpur Metropolitan City in the Chitwan district, excluding ward no 14,15 and 16. Necessary adjustments were made accordingly while facilitating accessibility by translating the questionnaire back and forth between Nepali and English languages.

Using a pre tested questionnaire the following information were collected. Socio demographic characteristics including age, ethnicity, religion, education, occupation and average monthly income. The questionnaire included knowledge related questions as knowledge on BPCR components, danger signs and symptoms during pregnancy, labour and postpartum, ANC visits.

The women were asked about obstetric factors, institutional factors and role of family and neighbors.

Operational definitions

Level of BPCR Practice: A woman was considered “well prepared” for birth and its complications when she practiced first four critical components among seven basic components **(i)** identified transportation for delivery, ii) saved money, iii) identified health facility, iv) identified blood donor v) identified skilled health provider, vi) identifying labour and vii) birth companion, preparing necessary materials for mother and baby).[25]

BPCR components knowledge: A woman was considered adequate knowledge on BPCR components when she spontaneously mentioned at least four components.

Knowledge on key danger signs during pregnancy: A woman was considered having adequate knowledge, if she spontaneously at least three danger signs: vaginal bleeding, swollen hands/face, blurred vision, severe abdominal pain, reduced/ no fetal movement, smelling discharge during pregnancy.

Knowledge on key danger signs during labor: A woman was considered having adequate knowledge, if she spontaneously at least three danger signs: severe vaginal bleeding, severe headache, prolonged labor (>12huors), convulsion, and retained placenta during labor/childbirth.

Knowledge on key danger signs during postpartum: A woman was considered having adequate knowledge, if she spontaneously at least three danger signs: severe vaginal bleeding, foul-smelling vaginal discharge, and high fever, unless considered as inadequate knowledge.

Data collection process

The data was collected through face-to-face interview during the immunization date in selected wards institution along with their out-reach clinic which was held from 9-13 of every month, before immunizing the child of respective mother only after getting written informed consent.

Data analysis

The collected data were coded, entered, cleaned and analyzed by IBM SPSS 20 version. Firstly, descriptive statistics, such as frequencies, percentages, and medians were computed to provide an overview of data. Practice level was divided into well prepared and less prepared where, those mothers who followed four critical components among seven were considered “well prepared” and the remaining were considered “less prepared”. Additionally, inferential statistics were employed, specifically the Pearson chi-square test at a significance level of 5%, to explore potential associations between level of practice and selected explanatory variables. A multi-collinearity diagnostic test was conducted to check for collinearity among the independent variables using VIF with the threshold of 10 and there was no multicollinearity detected between the variables.

Data quality control

To maintain the validity of the measurement, questions were drawn from the previously validated survey and developed based on an extensive literature search with frequent review to enhance their quality. The questionnaire underwent a pre-testing phase in other ward of Bharatpur Metropolitan City in the Chitwan district, excluding ward no 14,15 and 16. Necessary adjustments were made

accordingly while facilitating accessibility by translating the questionnaire back and forth between local language (Nepali) and English.

Ethical considerations

Ethical approval was obtained from Thesis Committee of the School of Public Health, Chitwan Medical College and Ethical Review Committee of Chitwan Medical College and from Chitwan Medical College Institutional Review Committee (IRC). (Ref: CMC-IRC/079/080-016). Letter of support was obtained from the health section of Bharatpur Metropolitan Office and respective health institutions before undertaking the study and written informed consent was obtained from the respondents before the interview. For Privacy and confidentiality, all interviews were conducted in private and all cautions were taken to ensure confidentiality. Participants was informed of their rights to deny participation in the study or to withdraw from the study at any stage of data collection. Respondents were provided with the necessary information on importance of birth preparedness and complication readiness to avoid delays in seeking and receiving health care services.

Results

Socio-demographic and obstetric Characteristics

A total of 220 recently delivered women were interviewed. The age range of respondents varied from 17 to 48, with median age of 26. By ethnicity 101(45.9%) were Brahmin/Chhetri and 4(1.8%) were Thakuri/Sanyasi. The major predominant religion includes Hindu 181(82.3%) and Christianity 7(3.2). Occupationally 169(76.8%) were housewives followed by services 10(4.5%). Regarding educational background, 56.6% had secondary education and 12.7% had bachelors and

above. The majority of respondents family income 80(36.4%) were between 30-50 thousand. Regarding the birth order, 48.2% of the women had already given birth for one child while 1(0.5%) had four children. About 215(97.7%) of respondents attended antenatal care in their recent pregnancy. Among those women who attended ANC more than four times were 104(47.3%) and 111(50.5%) less than four visits (Table 1).

Table 1: Socio-demographic and obstetric characteristics of recently delivered women, Bharatpur, Chitwan, Nepal (n=220)

Characteristics	Frequency	Percent (%)
**Age**		
17-25	109	49.5
>25	111	50.5
Median/IQR=26/6, Min/Max=17,48		
**Family Type**		
Nuclear	116	52.7
Joint/Extended	104	47.3
**Ethnicity**		
Brahmin/Chhetri	101	45.9

Janajati	66	30
Dalit	42	19.1
Madhesi	7	3.2
Thakuri/Sanyasi	4	1.8
**Religion**		
Hindu	181	82.3
Buddhism	32	15.5
Christianity	7	3.2
**Educational level (n=215)**		
General literate	6	2.7
Basic	56	25.9
Secondary	125	56.6
Bachelors and above	28	12.7
**Mother's occupation**		
Housewife	169	76.8
Agriculture	20	9.1
Business	17	7.7
Services	10	4.5

Labor	2	0.9
Others*	2	0.9
**Monthly income level**		
Less than 10,000	12	5.5
10,000-30,000	88	40
30,000-50,000	80	36.4
More than 50,000	40	18.2
**Birth order**		
One	106	48.2
Two	94	42.7
Three	19	8.6
Four	1	0.5
**Place of delivery**		
Institutional	217	98.6
Home	3	1.4
**ANC visit**		
Yes	215	97.7
No	5	2.3

**Number of ANC (n=215)**		
Less than four times	111	50.5
Greater than or equal to four	104	47.3
**Complication experience**		
Yes	101	45.9
No	119	54.1
Complications (101)		
Smelling discharge	22	22.2
Convulsions	7	7.1
Swollen hands and face	29	29.3
Labor lasting more than 12 hours	7	7.1
Severe headache	13	13.1
Vaginal bleeding	22	22.2
Blurring of vision	2	2
Abdominal pain	38	43.3
Others**	17	17.2

Other*student, other** vomiting, allergy, diabetes, meconium aspiration syndrome, High blood pressure.

Participants Knowledge about key danger signs

Knowledge regarding the components of Birth Preparedness and Complication Readiness (BPCR), 59.1% of women had adequate knowledge. Fig 1 Bar graph depicting the distribution of women's knowledge regarding danger signs associated with pregnancy, childbirth, and the postpartum period. The blue bars represent the percentage of women who demonstrated adequate knowledge by identifying at least three key danger signs: 50.9% for pregnancy, 20.5% for childbirth, and 27.7% for the postpartum period. Conversely, the orange bars indicate the percentage of women with inadequate knowledge, having mentioned fewer than three danger signs: 49.1% for pregnancy, 79.5% for childbirth, and 72.3% for the postpartum period. When asked about knowledge on ANC checkups, 90.5% of women had knowledge about it while 9.5% did not.

Table 2 outlines the most commonly mentioned items of danger signs during pregnancy were vaginal bleeding 131(65.2%), severe headache 80(39.8%) and swollen hand and face 69(34.3%). During labor and delivery, the majority of listed was severe bleeding 155(86.1), severe headache 68(37.8%) and loss of consciousness 61(33.9%). Similarly, severe bleeding 171(89.1%) was the most commonly mentioned items.

Table 2. Knowledge of respondents on danger signs during pregnancy, labour and post-partum period in Bharatpur, Chitwan, Nepal (n=220)

**Danger sings during pregnancy**	**Frequency**	**Percentage (%)**
Vaginal bleeding	131	65.2
Severe headache	80	39.8
Swollen hand/face	69	34.3
Blurring of vision	36	17.9

Severe lower abdominal pain	134	66.7
Smelling discharge	86	42.8
Convulsion	15	7.5
Reduced/no fetal moment	20	10
**Danger signs during labor/childbirth**		
Severe bleeding	155	86.1
Severe headache	68	37.8
Loss of consciousness	61	33.9
Labor lasting >12 hours	56	31.1
Placenta not delivered 30 min after baby birth	32	17.8
**Danger signs during Postpartum**		
Severe bleeding	171	89.1
Severe headache	72	37.5
Convulsion	45	23.4
High fever	92	47.9
Foul vaginal discharge	24	12.5

Institutional related factors

Table 3 describes the distance to health and delivery facilities, as well as the availability of 24-hour delivery services with median distance to health facilities was 15 minutes, minimum of 2 minutes and a maximum 35 minutes. The majority of participants reported having access to a

health facility within 20 minutes. However, over 80% of participants reported a distance of more than 30 minutes to delivery facilities. Additionally, 61.8% of participants reported that 24-hour delivery services were not available. For those whose nearest health facility did not have delivery/24 hours services, 82.3% of respondents reported that their nearest delivery facility was more than 30 minutes away.

Table 3: **Institutional related information among recently delivered women in Bharatpur, Chitwan, Nepal (n=220)**

**Variables**	**Frequency**	**Percentage (%)**
**Distance to health facilities**		
Less than 20 min	168	76.4
More than 20 min	52	23.6
Median/IQR=15/10 MIN, MAX=2, 35		
**Availability of delivery/24hrs facility**		
Yes	84	38.2
No	136	61.8
**Distance to delivery facilities**		
Less than 30 min	39	17.7
More than 30 min	181	82.3

Table 8 presents the support received by the women during delivery from their family and neighbors. The first variable is the decision maker for delivery, which refers to who makes the decision about the place of delivery. The frequency of women who made the decision alone was

43, accounting for 19.5% of the total sample. The frequency of women who made the decision with their husbands was 59, accounting for 26.8% of the total sample. The frequency of women who made the decision jointly with their family members was 105, accounting for 47.7% of the total sample. The frequency of women whose family members alone made the decision was 13, accounting for 5.9% of the total sample.

When asked whether they received assistance from community people, 38.6% of the respondents that they did, while 61.4% did not. For those who did receive assistance, the most common types of assistance provided were arranging transportation (60.2%) and arranging money (32.5%). Only 3.6% of respondents receiving assistance with arranging blood, while 24.1% reported receiving other types of assistance.

Family and neighbors support related information

Table 4 presents the support received by the women during delivery from their family and neighbors. About 19.5% of women who made the decision alone,26.8% made with their husbands while 5.9% responds that family members alone made decision for delivery. When asked whether they received assistance from community people, 38.6% of the respondents that they did, while 61.4% did not. For those who did receive assistance, the most common types of assistance provided were arranging transportation (60.2%), arranging money (32.5%), arranged blood and 24.1% reported receiving other types of assistance.

Table 4: Support by family and neighbors related information among recently delivered women in Bharatpur, Chitwan, Nepal (n=220)

Variables	Frequency	Percentage (%)

**Decision maker for delivery**		
Women alone	43	19.5
Women and husband	59	26.8
Jointly	105	47.7
Family members alone	13	5.9
**Assistant by community people**		
Yes	85	38.6
No	135	61.4
**If yes, what are those things (n=85)**		
Arrange transportation	50	60.2
Arrange money	27	32.5
Arrange blood	3	3.6
Others*	20	24.1

**denotes multiple response: food, cloths*

Birth Preparedness practices

Fig 2 bar graph illustrating various aspects of childbirth preparedness among women. The orange bars indicate the proportion of women who took specific preparatory actions: 91.4% identified the place of delivery, 97.7% saved money for childbirth, 87.3% arranged transportation, 85.9% selected a companion, and 90% procured necessary materials. In contrast, 52.3% identified skilled birth attendants, and only 36.4% made preparations for blood donors, highlighting a significant gap in blood donor preparedness compared to other aspects of childbirth readiness. (Table 5)

Table 5: Birth preparedness and complication readiness practice among recently delivered women in Bharatpur, Chitwan, Nepal (n=220)

Practices BPCR Components	Frequency	Percentage (%)
**Identified place of delivery**		
Yes	201	91.4
No	19	8.6
**Identified skilled birth attendant**		
Yes	115	52.3
No	105	47.7
**Saved money for childbirth**		
Yes	215	97.7
No	5	2.3
**Arranged Transportation**		
Yes	192	87.3
No	28	12.7
**Identified blood donor**		
Yes	80	36.4
No	140	63.6
**Identified companion**		
Yes	189	85.9
No	31	14.1

Arranged necessary materials		
Yes	198	90
No	22	10

Level of BPCR practice

Fig 3 Pie chart depicting the distribution of women's birth preparedness and complication readiness scores. The blue segment represents 103 women (46.8%) who were classified as well-prepared for birth and potential complications, based on key components of birth preparedness and complication readiness. The orange segment represents 117 women (53.2%) who were categorized as less prepared. This distribution highlights the proportion of women with varying levels of preparedness for childbirth and associated complications. (Table 6).

Table 6: Level of Birth Preparedness and Complication Readiness Practices among recently delivered woman in Chitwan, Nepal (n=220)

Level of BPCR Practiced	Frequency	Percentage (%)
Well Prepared	103	46.8
Less Prepared	117	53.2
**Total**	**220**	**100**

Association between level of BPCR Practice and some explanatory variables

In this study, the chi-square test was employed to analyze the relationship between the independent variables and the level of preparedness. The results indicated that significant differences in the level of preparedness were observed concerning family type (P=0.025), knowledge on components of BPCR (P= 0.001), knowledge on signs and symptoms during pregnancy (P=0.002), knowledge on signs and symptoms during labor/childbirth (P-value=0.008), knowledge on signs and symptoms during postpartum (P=0.05) and knowledge on ANC visit as protocol (P= 0.02) (Table 7). However, no significant differences were found in level of preparedness based on age, occupational status, religion, birth order,

Table 7: Association between level of BPCR practices and other explanatory variables among recently delivered woman in Chitwan, Nepal (N=220)

Variables	Level of BPCR practice		Chi-square	p-value
	Well prepared %	Less prepared %		
**Age of Child**				
0-4	47 (50)	47 (50)	3.596	0.166
5-9	21 (36.2)	37 (63.8)		
10-12	35 (51.5)	33 (48.5)		
**Mothers age**				
17-25	46 (42.2)	63 (57.8)	1.849	0.174
>25	57 (51.4)	54 (48.6)		
**Family type**			5.057	
Nuclear	46 (39.7)	70(60.3)		**0.025***
Joint/ Extended	103 (46.8)	47 (45.2)		

**Ethnicity**				
Brahmin/Chhetri	50 (49.5)	51 (50.5)	0.541	0.462
Non-Brahmin/Chhetri	53 (44.5)	66 (55.5)		
**Religion**				
Hindu	84 (46.4)	97 (53.6)	0.069	0.793
Non-Hindu	19 (48.7)	20 (51.3)		
**Mother education (n=215)**				
General/basic	27 (43.5)	35 (56.5)	0.663	0.415
Secondary and above	76 (49.7)	77 (50.3)		
**Mother occupation**				
Housewife/agriculture	85 (45)	104 (55)	1.833	0.176
Other than housewife	18 (58.1)	13 (41.9)		
**Monthly income**				
Less than 50,000	41 (41)	59 (59)	2.493	0.114
More than 50,000	62 (51.7)	58 (48.3)		
**Age at marriage**				
Teenage (≤19)	36 (45)	44 (55)	0.167	0.683
Above teenage (>19)	67 (47.9)	73 (52.1)		
**Knowledge on components of BPCR**				
Adequate	83 (63.8)	47 (36.2)	37.00	**<0.001***
Inadequate	20 (22.2)	70 (77.8)		

**Knowledge on signs and symptoms during pregnancy**				
Adequate	64 (57.1)	48 (42.9)	9.768	**0.002***
Inadequate	39 (36.1)	69 (63.9)		
**Knowledge on signs and symptoms during childbirth**				
Adequate	29 (57.1)	16 (42.9)	7.059	**0.008***
Inadequate	74 (36.1)	101 (63.9)		
**Knowledge on signs and symptoms during postpartum**				
Adequate	35 (57.4)	26 (42.6)	3.779	**0.05***
Inadequate	68 (42.8)	91 (57.2)		
**Knowledge on ANC visit protocol**				
Yes	100 (50.3)	99 (49.7)	9.868	**0.02***
No	3 (14.3)	18 (85.7)		
**Times of child bear**				
≤2	91 (45.5)	109 (54.5)	1.535	0.215
>2	12 (60)	8(40)		
**ANC visit**				

Yes	103 (47.9)	112 (52.1)		0.062$^{\beta}$
No	0 (0)	5 (100)		
**Number of ANC visit**				
Less than four times	6 (24)	19 (76)	5.535	**0.019***
Greater than or equal to four times	93 (48.9)	97 (51.1)		
**Place of delivery**				
Home	0 (0)	3 (1.4)		0.250 $^{\beta}$
Institutional	103 (47.5)	114 (51.8)		
**Complication experiences**				
Yes	48 (47.5)	53 (52.8)	0.037	0.847
No	55 (46.2)	64 (53.8)		

**Denotes significant association (By applying Pearson chi-square test at 5% level of significance) and β denotes association by applying Fisher's Exact test at 5% level of significance.*

Test of multicollinearity between independent variables and Birth preparedness and complication readiness (BPCR)

Table 8 presents that, those independent variables which where statistically significant and associated with birth preparedness and complication readiness practices were further used to test the multicollinearity among those independent variables.

Table 8: Test of multicollinearity between independent variables and BPCR practice among recently delivered woman in Chitwan, Nepal (n=220)

Independent variables	Tolerance	VIF (Variance Inflation Factor
Family type	0.978	1.023
Knowledge on components of BPCR	0.816	1.226
Knowledge on key danger signs during pregnancy	0.715	1.399
Knowledge on key danger signs during labor	0.611	1.636
Knowledge on key danger signs during post-partum	0.584	1.711
Number of ANC visits	0.930	1.075

Multivariate analysis of factors associated with BPCR practice

Six characteristics that exhibit statistically significant association ($p<0.05$) with birth preparedness and complication readiness practice at 95% CI during bivariate analysis were further subjected to multivariate logistic regression for adjustment of possible confounder. Multicollinearity test was done. None of them have tolerance <0.1 and VIF >10. There was no problem of serious collinearity among independent variables.

Birth preparedness practice was found to be statistically significant ($p<0.05$) with knowledge on components of BPCR. Women with adequate knowledge on components of BPCR are 5 times

more likely to practice or well prepared for birth and complication as compared to women with inadequate knowledge (AOR=5.34,95%CI: (2.728-10.478). Table 9

Table 9: Multivariate analysis of factors associated with BPCR practice among recently delivered woman in Chitwan, Nepal (n=220)

**Variable**	**COR (95% CI)**	**AOR (95%CI)**	**p-value**
**Family Type**			
Nuclear	*Ref*	*Ref*	0.88
Joint/Extended	1.846(1.080-3.155)	1.689(0.926-3.080)	
**Knowledge on components of BPCR**			
Inadequate	*Ref*	*Ref*	**0.00**
Adequate	6.181(3.351-11.402)	**5.347(2.728-10.478) ***	
**Knowledge on key danger signs during pregnancy**			
Inadequate	*Ref*	*Ref*	
Adequate	2.359(1.371-4.058)	1.332(0.669-2.652)	0.414
**Knowledge on key danger signs during labor**			
Inadequate	*Ref*	*Ref*	0.675
Adequate	2.474(1.253-4.883)	1.213(0.492-2.991)	
**Knowledge on key danger signs during postpartum**			

Inadequate	*Ref*	*Ref*	
Adequate	1.801(0.992-3.272)	0.801(0.346-1.856)	0.605
**Knowledge on ANC visit as**			
Less than four times	*Ref*	*Ref*	
Greater than four times	2.499(1.451-4.305)	2.151(1.170-3.954)	0.14

*Variables significant at p<0.05 *Ref*: Reference Category CI: Confidence Interval OR: Odds Ratio

Discussions

BPCR is one of the proven and effective health care strategy in preventing maternal mortality especially for countries with prevailing high risk of maternal deaths and inefficient health care system. The study found that the overall prevalence of BPCR was 46.8%, which is comparable to rates reported in studies conducted in Southern Nigeria (48.4%)[33] and Southern Ethiopia (48.5%)[10], Sidama (17%), Wolyita (18.3%) zones of Ethiopia[34, 35]. However, the prevalence rate was better than that reported in a study conducted in Dang district (44.36%)[21]. On the other hand, the rate was lower than rates reported in studies conducted in Rapti municipality, Kaski district[20, 36] and other South Asian countries, including Sri Lanka, Karnataka, India, and West Bengal[37, 38], as well as Southwest Nigeria and Tanzania[16, 39]. The most common birth preparedness practice observed in this study was saving money, which may be explained by the fact that both women and their partners know that money is required to facilitate referral in case of complications. And it is interesting to note that other studies on birth preparedness have reported similar findings[40, 41]. The study found that identification of blood donor as an obstetric

emergency was not considered crucial issue by some respondents as other components where only 34% identified potential blood donor. However, other study reported even lower percent of respondents practiced this component[9]. The variation in findings may be attributed to several factors, such as differences in the study population, with our study involving recently delivered women, while the studies in South Ethiopia[34] and Dang[42] included pregnant women and both delivered and pregnant women, respectively. Pregnant women may be unable to report their preparedness for situations they have not yet experienced. Additionally, variations in female literacy levels, empowerment, study setting, socio-cultural difference, and methodological differences in assessing BPCR could also contribute to the differences in findings.

Nearly three-quarters (72.1%) and over one-third (45.3%) of the study were knowledgeable about identifying place of delivery and arranging transportation as essential components of BPCR, this aligns with similar findings reported in other studies. In a survey conducted in Samara Logia Town, where 75% and 45% of participants were knowledgeable on those components[43]. The study revealed that 59.1%, 50.9%, of participants had knowledge about the components of BPCR and danger signs during pregnancy This finding is in contrast to a study conducted in Farta district, Ethiopia, which reported a significantly lower percentage of 14.3% and 23.3% of participants with knowledge of the components and danger signs during pregnancy respectively[11]. The study revealed that the participants' understanding of danger signs during labor/childbirth and the postpartum period was inadequate, with only 20.5% and 27.3% of them reporting familiarity with these signs, respectively which is consistent with the study done in Sodo town, Southern Ethiopia,[10]The finding that severe bleeding was identified as a major sign and symptom during childbirth is similar in both the current study and a study conducted in Mizan-Aman, Southwest Ethiopia[39]. Women having knowledge of obstetric danger signs might have chance to improve

their decision-making ability on their maternal and child health care service utilization and can respond immediately in the event of emergencies to avoid delays[11].

Approximately Ninety Eight Percent of the women have had at least one ANC visit, about half (47.3%) attended the minimum recommended number of four or more visits, was found to be low as compared to the national NDHS data (81%)[44] and studies in Kassena- Nankana of Ghana[45]. This study included a specific decision-making variable which is absent from most studies. But in this study almost half of them made decision jointly regarding the place of delivery as in the study in Rapti municipality[32].

Our study identified that the level of BPCR practice is significantly associated with family type (χ^2=5.057, p=0.025)and knowledge on ANC visit as protocol (χ^2=9.868, p=0.02) which is compliance with the findings done in Kenya and Tanzania and Rapti Municipality of Chitwan, Nepal[32, 46]. Similarly, knowledge on components of BPCR ((χ^2=37, p=0.001), danger sign and symptoms during pregnancy (χ^2=9.768, p=0.002), labor/childbirth and postpartum (χ^2=3.779, p=0.05) are significantly associated with BPCR practice, which is similar with the findings of community- based study done in rural area in Bangladesh, Mizan-Aman Town, Southwest Ethiopia and Nepal where association between knowledge and practice was observed[6, 22]. Also, the current study found that the BPCR practice was statistically significant with number of ANC visits. This finding is supported by the study in central Tanzania where woman who had attended 4 or more times antenatal care were better prepared than women who had attended <4 times antenatal care[47]. The significant influence of woman’s literacy level on BPCR practice is highlighted by many studies from Sri-lanka and African countries[48]. However, in our study and a study in Biratnagar, Nepal, Karnataka India, Bangladesh, woman’s education level did not show

a significant association with BPCR[15, 40, 49]. This could be attributed to overall high (97.7%) literacy rate in this study population.

Having the knowledge of BPCR increases the practice of BPCR among recently delivered women and 5 times more likely to be well prepared according to this study. This is comparable with the studies from Central Ethiopia [50] Bench Maji zone [51] and Farta district [11]. The inference of this finding could be once women become knowledgeable about BPCR, they are expected to practice the components of BPCR and ready to act upon it when it occurs.

The advantage of asking women who are recently delivered about the birth preparedness as they have completed their pregnancies and already made all necessary arrangement. As there may be variation in their service use in pregnant women, they will not provide the information based on their plan to use those services.

Conclusions

Although not satisfactory in the view of expectations, a relatively higher practice of BPCR had been observed in the study area compared to other reports. Majority of participants saved money as one of preparation for childbirth while only few prepared blood donors in case of obstetric complication and emergency. This study also revealed that the respondent's knowledge of danger sign and symptoms during pregnancy was adequate but during child birth and postpartum was low. Similarly, the women having recommended ANC visit was low as compared to national data. These findings lend support to reinforce health education on BPACR during pregnancy. Further the principal factors affecting the BPCR practice were family type, ANC visit, knowledge on components of BPCR, danger signs during pregnancy, labor/Childbirth, and postpartum period,

which suggests more intervention to be taken at health institution and health care providers. Furthermore, pregnant women should continuously be encouraged to attend ANC as per the protocol so that interventions such as Sensitization of pregnant women, during ANC visit by the health workers, regarding Components of BPCR, danger sign and symptoms during pregnancy, may enhance BPCR.

Study Limitations

- Self-reported practice may compromise the validity of the findings.
- There could also be information bias as the questions were asked to all those who delivered in the last 12 months. Some might not have recalled the danger signs, and symptoms.

Acknowledgements

We would like to thank school of public health, Chitwan Medical college, health section of Bharatpur metropolitan city and health institutions for providing support in undertaking this research.

S1 File. SPSS dataset BPCR.

https://figshare.com/s/3199a871d0bdeae608e3

DOI: [10.6084/m9.figshare.26213132]

References

[1] Nations U. Targets and Strategies for Ending Preventable Maternal Mortality consensus statement.

[2] Mgawadere F, Kana T, Van Den Broek N. Measuring maternal mortality: A systematic review of methods used to obtain estimates of the Maternal Mortality Ratio (MMR) in low- and middle-income countries. *Br Med Bull* 2017; 121: 121–134.

[3] United Nations . Sustainable Development Goals. GenevaWorld Health Organization, 2015., http://www.un.org/sustainabledevelopment/sustainable-development-goals/.

[4] Basnet I. National Safe Motherhood Plan (2002-2017). 2002; 1–23.

[5] Ihomba PK, Nyamari JM, Were T. Birth Preparedness and Complication Readiness among Women with Preg-. *East African Heal Res J* 2020; 4: 26–32.

[6] Moinuddin M, Christou A, Hoque DME, et al. Birth preparedness and complication readiness (BPCR) among pregnant women in hard-to-reach areas in Bangladesh. *PLoS One* 2017; 12: 1–19.

[7] Yılmaz S, Calikoglu EO, Kosan Z. Several studies across the world suggest that promoting BPACR interventions improves preventive behaviours, improves knowledge of

mothers about danger signs and leads to improvement in care- seeking during obstetric emergency. *Niger J Clin Pract* 2019; 22: 1070–1077.

[8] Zepre K, Kaba M. Birth preparedness and complication readiness among rural women of reproductive age in Aabeshige district, Gguraghe zone, SNNPR, Eethiopia. *Int J Womens Health* 2017; 9: 11–21.

[9] Affipunguh PK, Laar AS. Assessment of knowledge and practice towards birth preparedness and complication readiness among women in Northern Ghana : a cross-sectional study. 2016; 2: 121–129.

[10] Azeze GA, Mokonnon TM, Kercho MW. Birth preparedness and complication readiness practice and influencing factors among women in Sodo town, Wolaita zone, Southern Ethiopia, 2018; Community based cross-sectional study. *Reprod Health*; 16. Epub ahead of print 29 March 2019. DOI: 10.1186/s12978-019-0703-z.

[11] Limenih MA, Belay HG, Tassew HA. Birth preparedness , readiness planning and associated factors among mothers in Farta district , Ethiopia : a cross-sectional study. 2019; 4: 1–10.

[12] Wudu MA, Tsegaye TB. Birth preparedness and complication readiness and associated factors among recently delivered mothers in mizan-aman town, Southwest Ethiopia, 2019. *Int J Womens Health* 2021; 13: 177–187.

[13] Pervin J, Tin Nu U, Rahman AMQ, et al. Level and determinants of birth preparedness and complication readiness among pregnant women: A cross sectional study in a rural area in Bangladesh. *PLoS One*; 13. Epub ahead of print 1 December 2018. DOI: 10.1371/journal.pone.0209076.

[14] Danna VA. Utility of the three-delays model and its potential for supporting a solution-based approach to accessing intrapartum care in low- and middle-income countries. A qualitative evidence synthesis.

[15] Moran AC, Sangli G, Dineen R, et al. Birth-preparedness for maternal health: Findings from Koupéla district, Burkina Faso. *J Heal Popul Nutr* 2006; 24: 489–497.

[16] Soubeiga D, Gauvin L, Hatem MA, et al. Birth Preparedness and Complication Readiness (BPCR) interventions to reduce maternal and neonatal mortality in developing countries : systematic review and meta-analysis. 2014; 1–11.

[17] Safe Motherhood Program in Nepal Evolution and Impact Public health in Nepal. Blogspot.com accessed at August 6 mondey.

[18] UNFPA Asiapacific | Maternal health, https://asiapacific.unfpa.org/en/maternal-health (accessed 5 August 2022).

[19] Sitaula S, Basnet T, Agrawal A, et al. Prevalence and risk factors for maternal mortality at a tertiary care centre in Eastern Nepal- retrospective cross sectional study. *BMC Pregnancy Childbirth* 2021; 21: 1–8.

[20] Marta Levitt-Dayal. Saving Mothers' Lives: What works, a field guide for implementing best practices in safe motherhood. 2002; 32–56.

[21] Ekabua JE, Ekabua KJ, Odusolu P, et al. Awareness of Birth Preparedness and Complication Readiness in Southeastern Nigeria. 2011. Epub ahead of print 2011. DOI: 10.5402/2011/560641.

[22] South Asia's Quest for Reduced Maternal Mortality: What the Data Show,

https://blogs.worldbank.org/health/south-asia-s-quest-reduced-maternal-mortality-what-data-show (accessed 5 August 2022).

[23] Nadeeka Perera SEG. Birth and emergency preparedness and associated factors among postnatal mothers at Base Hospital Balangoda, Sri Lanka.

[24] Ministry of Health N, 2017. l; NE and I. Nepal Demographic and Health Survey 2016. Kathmandu, Nepal: Ministry of Health, Nepal. *Eugen Q* 1967; 14: 54–59.

[25] Mukhopadhyay DK, Mukhopadhyay S, Bhattacharjee S, et al. Status of birth preparedness and complication readiness in Uttar Dinajpur District, West Bengal. *Indian J Public Health* 2013; 57: 147–154.

[26] Bintabara D, Mohamed MA, Mghamba J, et al. Birth preparedness and complication readiness among recently delivered women in chamwino district, central Tanzania: A cross sectional study 'Obstetrics'. *Reprod Health*; 12. Epub ahead of print 16 May 2015. DOI: 10.1186/s12978-015-0041-8.

[27] 10057-Article Text-51571-1-10-20080827.

[28] Osaze D, Efegbere HA, Akwa OP. Correlates of birth preparedness practices and complication readiness among women in eight communities in Delta State, Nigeria Daniel. 2021; 244–252.

[29] Saaka M, Alhassan L. Prevalence and predictors of birth preparedness and complication readiness in the Kassena-Nankana district of Ghana: An analytical cross-sectional study. *BMJ Open* 2021; 11: 1–9.

[30] Bal Kumar KC. Ministry of Health and Population (MOHP) [Nepal], New ERA, and ICF

International Inc. 2012. Nepal Demographic and Health Survey 2011. Kathmandu, Nepal: Ministry of Health and Population, New ERA, and ICF International, Calverton, Maryland. *Asian Pacific Migr J* 2008; 17: 287–309.

[31] NSO. *National Population and Housing Census 2021*. 2021.

[32] Silwal K, Poudyal JK, Shah R, et al. Factors Influencing Birth Preparedness in Rapti Municipality of Chitwan, Nepal. *Int J Pediatr (United Kingdom)*; 2020. Epub ahead of print 2020. DOI: 10.1155/2020/7402163.

[33] Mcpherson RA, Khadka N, Moore JM, et al. Are Birth-preparedness Programmes Effective? Results From a Field Trial in Siraha District, Nepal. *J Heal Popul NUTR* 2006; 24: 479–488.

[34] Hailu M, Gebremariam A, Alemseged F, et al. Birth preparedness and complication readiness among pregnant women in Southern Ethiopia. *PLoS One*; 6. Epub ahead of print 2011. DOI: 10.1371/journal.pone.0021432.

[35] Gebre M, Gebremariam A, Abebe TA. Birth preparedness and complication readiness among pregnant women in Duguna Fango District, Wolayta Zone, Ethiopia. *PLoS One* 2015; 10: 1–12.

[36] Health J, Nutr P. Birth Preparedness and Complication Readiness among Slum Women in Indore City, India. 2010; 28: 383–391.

[37] Trends in maternal mortality: 2000 to 2017: estimates by WHO, UNICEF, UNFPA, World Bank Group and the United Nations Population Division. Geneva: World Health Organization; 2019.

[38] Health topics, https://www.who.int/health-topics/#M (accessed 4 August 2022).

[39] Mahadevia G, Vikas M. International Institute for Population Sciences (IIPS) and ICF. 2017. National Family Health Survey (NFHS-4), 2015-16: India. Mumbai: IIPS. *Int Sci J Environ Sci* 2012; 21: 7–15.

[40] Akshaya KM, Shivalli S. Birth preparedness and complication readiness among the women beneficiaries of selected rural primary health centers of Dakshina Kannada district, Karnataka, India. *PLoS One*; 12. Epub ahead of print 1 August 2017. DOI: 10.1371/journal.pone.0183739.

[41] Afik A. The relationship between response time and patient survival with emergency treatment by the Code Blue Team. *Indian J Public Heal Res Dev* 2018; 9: 1803–1807.

[42] Bhusal CK, Bhattarai S. INVOLVEMENT OF MALE IN BIRTH PREPAREDNESS IN TULSIPUR MUNICIPALITY OF DANG DISTRICT NEPAL, www.jcmc.cmc.edu.np (2015).

[43] Estimates by WHO, UNICEF, UNFPA WBG and the UNPD. TRENDS IN MATERNAL MORTALITY2000 to 2017.

[44] Debelew GT, Afework F, Yalew AW. Factors affecting birth preparedness and complication readiness in Jimma Zone, Southwest Ethiopia: A multilevel analysis. *Pan Afr Med J*; 19. Epub ahead of print 11 November 2014. DOI: 10.11604/pamj.2014.19.272.4244.

[45] Florence M, Atuhaire C, Nkfusai CN, et al. Knowledge and practice of birth preparedness and complication readiness among pregnant women attending antenatal clinic in openzinzi

hciii, adjumani district, Uganda. *Pan Afr Med J* 2019; 34: 1–9.

[46] Orwa J, Gatimu SM, Mantel M, et al. Birth preparedness and complication readiness among women of reproductive age in Kenya and Tanzania: a community-based cross-sectional survey. *BMC Pregnancy Childbirth*; 20. Epub ahead of print 1 December 2020. DOI: 10.1186/s12884-020-03329-5.

[47] Ibadin SH, Adam VY, Adeleye OA, et al. Birth preparedness and complication readiness among pregnant women in a rural community in Southern Nigeria. *S Afr J Obstet Gynaecol* 2016; 22: 47–51.

[48] Rodrigo CH, Kumarapeli V. Birth preparedness, complication readiness and associated factors among pregnant women seeking antenatal care at a Medical Officer of Health (MOH) area in Sri Lanka. *J Postgrad Inst Med* 2019; 6: 84.

[49] Subedi S. Knowledge and Practice on Birth Preparedness and Complication Readiness among Pregnant Women in Selected Ward of Biratnager Municipality, Nepal. *Tribhuvan Univ J* 2019; 33: 53–66.

[50] Girma D, Waleligne A, Dejene H. Birth preparedness and complication readiness practice and associated factors among pregnant women in Central Ethiopia, 2021: A cross-sectional study. *PLoS One* 2022; 17: 1–16.

[51] Gudeta TA, Regassa TM. Factors Associated with Birth Preparedness and Complication Readiness Among Pregnant Women in Bench Maji Zone, Southwest Ethiopia: A Community-Based Cross-Sectional Study. *Ethiop J Health Sci* 2019; 29: 567–576.

15 September 2024

Dr. Adaoha Pearl Agu, MBBS,

Academic Editor

PLOS ONE

Dear Dr. Agu,

Thank you for your thoughtful review of our manuscript titled "**Birth Preparedness and Complication Readiness Knowledge and Practices Among Recently Delivered Women: A Cross-Sectional Study In Bharatpur, Chitwan, Nepal**" (Manuscript ID: PONE-D-24-29050). We appreciate the time and effort the reviewers and editorial team have invested in evaluating our work. We are grateful for the constructive feedback and are pleased to submit our revised manuscript in response to the comments and suggestions provided. Below, we address each point raised during the review process:

1. **Discussion of Study Limitations (Publication Criterion 7)**

Reviewer Comment: Discuss the study limitations on the effects of the study outcomes to fully satisfy PLOS ONE's publication criteria number 7.

Response: We have revised the Discussion section of our manuscript to include a more comprehensive analysis of the study limitations. We acknowledge that while our study provides valuable insights, it is not without limitations. Specifically, we have expanded our discussion on the following points:

- **Recall Bias:** There could also be information bias as the questions were asked to all those who delivered in the last 12 months. Some might not have recalled the danger signs, and symptoms.
- **Validity of Findings:** Self-reported practice may compromise the validity of the findings.

The revised Discussion section now provides a clearer understanding of these limitations.

Grant Information and Financial Disclosure

Reviewer Comment: The grant information provided in the 'Funding Information' and 'Financial Disclosure' sections do not match.

Response: We have corrected the discrepancies between the 'Funding Information' and 'Financial Disclosure' sections. Our research was supported by a grant from our research grant committee. However, the committee did not provide specific grant numbers for this funding. Consequently, we are unable to include grant numbers in the 'Funding Information' section. We have clearly indicated this in the revised manuscript.

2. **Data Sharing and Accessibility**

Reviewer Comment: Explain ethical or legal restrictions on sharing data, or if there are none, provide a minimal anonymized data set.

Response:

No Restrictions: As there are no restrictions on data sharing, we have uploaded the anonymized data set to Figshare (https://figshare.com/s/3199a871d0bdeae608e3) [10.6084/m9.figshare.26213132]. The Data Availability statement in the manuscript has been updated accordingly.

3. **References to Figures**

Reviewer Comment: Ensure figures are correctly referenced in the text.

Response: We have updated the manuscript to correctly refer to Figures 2 and 3 in the text as required.

4. **Supporting Information Captions**

Reviewer Comment: Include captions for Supporting Information files and update in-text citations.

Response: Captions for all Supporting Information files have been added to the end of the manuscript. In-text citations have been updated to match the revised Supporting Information sections.

5. **Issues with Supporting Information File**

Reviewer Comment: The Supporting Information file [FInal data 101.sav] could not be opened.

Response: We have revised the Supporting Information file and re-uploaded it. The file is now accessible and has been confirmed to be properly formatted.

We appreciate the opportunity to revise our manuscript and believe that the changes made address the reviewers' concerns effectively. We have uploaded the revised manuscript, the marked-up version with track changes, and our response letter as requested.

Thank you for considering our revised manuscript. We look forward to your feedback.

Kind regards,

Asmita Ghimire

Chitwan Medical College, Tribhuwan University, Nepal

+9779866615678

ghimireasmita570@gmail.com

16 September 2024

Subject: Re: Manuscript PONE-D-24-29050R1 – Revisions and Resubmission

Dear Dr. Delas Alas Vida,

Thank you for your email regarding our manuscript entitled "Birth Preparedness and Complication Readiness Knowledge and Practices Among Recently Delivered Women: A Cross-Sectional Study in Bharatpur, Chitwan, Nepal" (Manuscript ID: PONE-D-24-29050R1).

We appreciate your feedback and are addressing the requested revisions as follows:

1. **Removal of Duplicate Files**: We have reviewed the files in our submission and removed all unnecessary or duplicate files. Only the relevant files pertaining to the current version of the manuscript have been included.
2. **Inclusion of Figure Legends**: We have updated the manuscript to include separate, detailed legends for each figure. The legends now provide clear and comprehensive descriptions to enhance the reader's understanding of the figures.
3. **Reference to Table 9**: We have ensured that Table 9 is properly referenced in the text of the manuscript. This reference is included to facilitate easy linking between the text and the table upon acceptance.

We have resubmitted the revised manuscript with the requested changes. Please let us know if any further adjustments are needed.

Thank you for your consideration and for providing us with the opportunity to revise our manuscript. We look forward to your feedback and hope for a favorable review.

Best regards,

Asmita Ghimire

Chitwan Medical College, Tribhuwan University, Nepal

ghimireasmita570@gmail.com

+9779866615678

Data Review URL

Click here to download Data Review URL

http://figshare.com/s/3199a871d0bdeae608e3
