# Supplementary material for: Birth preparedness and complication readiness knowledge, practices and its associated factors among recently delivered women: A cross-sectional study in Bharatpur, Chitwan, Nepal
Source: PLoS One. 2026 Jan 7;21(1):e0340376. doi: 10.1371/journal.pone.0340376 (PMC12779156; doi:10.1371/journal.pone.0340376)
Supplement: Attachment — Submitted filename: Response To Reviewers..docx [file pone.0340376.s002.docx]

16 September 2024

**Subject: Re: Manuscript PONE-D-24-29050R1 – Revisions and Resubmission**

Dear Dr. Delas Alas Vida,

Thank you for your email regarding our manuscript entitled “Birth Preparedness and Complication Readiness Knowledge and Practices Among Recently Delivered Women: A Cross-Sectional Study in Bharatpur, Chitwan, Nepal” (Manuscript ID: PONE-D-24-29050R1).

We appreciate your feedback and are addressing the requested revisions as follows:

1. **Removal of Duplicate Files**: We have reviewed the files in our submission and removed all unnecessary or duplicate files. Only the relevant files pertaining to the current version of the manuscript have been included.
2. **Inclusion of Figure Legends**: We have updated the manuscript to include separate, detailed legends for each figure. The legends now provide clear and comprehensive descriptions to enhance the reader's understanding of the figures.
3. **Reference to Table 9**: We have ensured that Table 9 is properly referenced in the text of the manuscript. This reference is included to facilitate easy linking between the text and the table upon acceptance.

We have resubmitted the revised manuscript with the requested changes. Please let us know if any further adjustments are needed.

Thank you for your consideration and for providing us with the opportunity to revise our manuscript. We look forward to your feedback and hope for a favorable review.

Best regards,

Asmita Ghimire

Chitwan Medical College, Tribhuwan University, Nepal

[ghimireasmita570@gmail.com](mailto:ghimireasmita570@gmail.com)

+9779866615678
