# Supplementary material for: Birth preparedness and complication readiness knowledge, practices and its associated factors among recently delivered women: A cross-sectional study in Bharatpur, Chitwan, Nepal
Source: PLoS One. 2026 Jan 7;21(1):e0340376. doi: 10.1371/journal.pone.0340376 (PMC12779156; doi:10.1371/journal.pone.0340376)
Supplement: Attachment — Submitted filename: Response_to_Reviewers_auresp_3.docx [file pone.0340376.s005.docx]

**17 September 2025**

**To**

**Adaoha Pearl Agu, MBBS, MSc, FMCPH
Academic Editor
PLOS ONE**

**Subject:** Rebuttal Letter for Manuscript (PONE-D-24-29050R1)- Birth Preparedness And Complication Readiness Knowledge And Practices Among Recently Delivered Women: A Cross-Sectional Study In Bharatpur, Chitwan, Nepal

**Dear Editor and Reviewers,**

Thank you for the opportunity to revise our manuscript entitled "[Birth Preparedness And Complication Readiness Knowledge And Practices Among Recently Delivered Women: A Cross-Sectional Study In Bharatpur, Chitwan, Nepal ]" (Manuscript ID: [PONE-D-24-29050R1]). We sincerely appreciate the time and effort you and the reviewers have invested in providing thoughtful and constructive feedback.

We have carefully considered all the comments and have revised the manuscript accordingly. Below, we provide a detailed point-by-point response to each comment. Reviewer comments are shown in **bold**, followed by our responses in regular text. All changes made in the manuscript are highlighted.

Reviewer #1 Comments and Author Responses

Abstract: We have revised the abstract’s background to include the rationale and purpose of the study.

The term “structured interview schedule” has been replaced with “structured interview questionnaire” in the Methods section.

The typographical error has been corrected.

The first sentence of results has been restructured for better clarity and readability.

We have added the specific proportion (46.8%) of women who were well prepared for birth and complications in the abstract.

**Main Manuscript**

### ****Introduction****

**Comment**: You need to check typos and grammatical errors.

**Response**: The introduction section has been carefully proofread and revised to correct all typographical and grammatical errors.

### ****Methods****

**Comment**: Justification for the study area is not available.

**Response**: We have added a justification for selecting Bharatpur Metropolitan City, citing its population size, diversity, and accessibility of maternal health services.

#### **Sample Size Calculation**

**Comment**: Any justification for considering 7% as margin of error instead of 5%? In the calculation, however, 6% is used.

**Response**. We have clarified that 6% was used as a practical compromise between statistical rigor and resource feasibility. This choice balanced sample size and field constraints while maintaining acceptable precision.

**Comment**: The paper you referenced stated that 29% lacked information on BPCR. Is this the same as the prevalence of poor practice?

**Response**: We acknowledge this distinction and have clarified in the text that we used the 29% as an approximate indicator due to the lack of direct prevalence data on BPCR practice in a similar setting. This limitation is now noted in the methodology.

**Comment**: How was the sample size distributed among wards and health institutions?

**Response**: We have added a description explaining that proportional allocation was used based on delivery caseloads of health facilities within selected wards.

#### **Measurements**

**Comment**: Any reference to the previously validated questionnaire?

**Response**: Yes, we have added references to the WHO and JHPIEGO tools from which the questionnaire was partially adapted.

**Comment**: Were there regular reviews of the questionnaire during data collection?

**Response**: We have now included a note that data collection was monitored regularly by the principal investigator, and weekly review meetings were held to address field challenges.

**Comment**: Study design needs to be written well and concisely.

**Response**: The study design section has been rewritten concisely as “A community-based descriptive cross-sectional study.”

**Comment**: The sampling of participants for the study should be clearly written.

**Response**: The sampling procedure has been revised to clearly explain how participants were selected .

**Comment**: Examples of obstetric and institutional factors investigated.

**Response**: Examples such as parity, place of delivery, and support received from health staff have been added to clarify these factors.

#### **Data Collection Process**

**Comment**: Data collection procedure needs to be written well/clearly.

**Response**: We have rewritten this section for clarity, detailing the steps followed during data collection.

**Comment**: There was no mention of who did the data collection and whether training was done before the data collection.

**Response**: Added. The data collection was carried out by the author, and data analysis was done with guidance from a university statistics professor. Pretesting of the tool were conducted prior to data collection.

#### **Data Analysis**

**Comment**: It would have been good to describe a bit more about scoring.

**Response**: We have elaborated on the scoring criteria used for BPCR components and knowledge levels.

### ****Results****

**Comment**: IQR is between 25th and 75th percentiles, not min and max.

**Response**: Corrected. **Comment**: Table 1 could have been divided into three parts.

**Response**: Revised as suggested. Table 1 is now split into three separate tables: Socio-demographic, Obstetric, and Complication Experience.

**Comment**: The tables need to be constructed well and referenced.

**Response**: All tables have been reconstructed for clarity and are now properly numbered and referenced in the manuscript.

**Comment**: Section on support received by women is repeated.

**Response**: The repeated section has been removed.

**Comment**: Chose Fig 2 or Table 5, Fig 3 or Table 6 – avoid duplication.

**Response**: We have retained the tables and removed the duplicate figures to avoid redundancy.

### ****Discussion****

**Comment**: Discussion repeats results and lacks implications.

**Response**: The discussion section has been revised to focus more on interpreting findings and drawing implications. We now include a specific section titled “Implications of the Findings,” summarizing key practical and policy takeaways.

### ****Language and Grammar****

**Comment**: The document needs a lot of work grammatically.

**Response**: The entire manuscript has undergone thorough grammar and language editing to improve clarity and academic tone.

We sincerely thank the reviewer for their helpful and detailed comments. We believe the revised manuscript has been significantly improved and now meets the journal’s standards. We are happy to make any additional changes if needed.

Sincerely,
**Asmita Ghimire**
On behalf of all co-authors
Chitwan Medical College, Tribhuwan University
ghimireasmita570@gmail.com
